# *N*-acetylglucosamine (GlcNAc) Triggers a Rapid, Temperature-Responsive Morphogenetic Program in Thermally Dimorphic Fungi

**DOI:** 10.1371/journal.pgen.1003799

**Published:** 2013-09-19

**Authors:** Sarah A. Gilmore, Shamoon Naseem, James B. Konopka, Anita Sil

**Affiliations:** 1 Department of Microbiology and Immunology and the Howard Hughes Medical Institute, University of California, San Francisco, San Francisco, California, United States of America; 2 Department of Molecular Genetics and Microbiology, Stony Brook University, Stony Brook, New York, United States of America; Duke University Medical Center, United States of America

## Abstract

The monosaccharide *N*-acetylglucosamine (GlcNAc) is a major component of microbial cell walls and is ubiquitous in the environment. GlcNAc stimulates developmental pathways in the fungal pathogen *Candida albicans*, which is a commensal organism that colonizes the mammalian gut and causes disease in the setting of host immunodeficiency. Here we investigate GlcNAc signaling in thermally dimorphic human fungal pathogens, a group of fungi that are highly evolutionarily diverged from *C. albicans* and cause disease even in healthy individuals. These soil organisms grow as polarized, multicellular hyphal filaments that transition into a unicellular, pathogenic yeast form when inhaled by a human host. Temperature is the primary environmental cue that promotes reversible cellular differentiation into either yeast or filaments; however, a shift to a lower temperature *in vitro* induces filamentous growth in an inefficient and asynchronous manner. We found GlcNAc to be a potent and specific inducer of the yeast-to-filament transition in two thermally dimorphic fungi, *Histoplasma capsulatum* and *Blastomyces dermatitidis*. In addition to increasing the rate of filamentous growth, micromolar concentrations of GlcNAc induced a robust morphological transition of *H. capsulatum* after temperature shift that was independent of GlcNAc catabolism, indicating that fungal cells sense GlcNAc to promote filamentation. Whole-genome expression profiling to identify candidate genes involved in establishing the filamentous growth program uncovered two genes encoding GlcNAc transporters, *NGT1* and *NGT2*, that were necessary for *H. capsulatum* cells to robustly filament in response to GlcNAc. Unexpectedly, *NGT1* and *NGT2* were important for efficient *H. capsulatum* yeast-to-filament conversion in standard glucose medium, suggesting that Ngt1 and Ngt2 monitor endogenous levels of GlcNAc to control multicellular filamentous growth in response to temperature. Overall, our work indicates that GlcNAc functions as a highly conserved cue of morphogenesis in fungi, which further enhances the significance of this ubiquitous sugar in cellular signaling in eukaryotes.

## Author Summary

In stark contrast to most fungal pathogens, thermally dimorphic fungal pathogens cause systemic infections in immunocompetent humans. Thermally dimorphic fungi grow in the soil as a multicellular filamentous form specialized for replication in this particular environmental niche. Upon infection of a human, these fungi transition to a parasitic cell type that is adapted for replication and pathogenesis within a mammalian host. In this work, we examined factors that are important for growth of the infectious, environmental form of thermally dimorphic fungi. We discovered that *N*-acetylglucosamine (GlcNAc), a ubiquitous carbohydrate with cellular roles across all kingdoms of life, stimulated a switch to the environmental form for two thermally dimorphic fungal pathogens, *Histoplasma capsulatum* and *Blastomyces dermatitidis*. Analysis of how fungal cells respond to GlcNAc revealed that these fungi possess two GlcNAc transporters that are important for controlling their ability to switch between infectious and parasitic states. Overall, our work begins to elucidate the pathways that promote growth in the infectious form of these organisms, which is critical to our understanding of environmental signals that promote disease transmission of thermally dimorphic fungi.

## Introduction

Cellular differentiation is an essential process for the development and growth of complex multicellular eukaryotic organisms. Similarly, many unicellular eukaryotic organisms undergo a program of cellular differentiation to produce a new cell type that is specialized for survival in a distinct environmental niche. In response to environmental stimuli, the family of thermally dimorphic fungal pathogens undergoes a program of cellular differentiation to transition between a saprophytic soil form and a parasitic host form [1]. The soil form is comprised of multicellular filaments that produce infectious spores. The parasitic form for the majority of thermally dimorphic fungi consists of a unicellular yeast form that is capable of evading host immune defenses. Temperature is the predominant environmental cue that promotes cellular differentiation of thermally dimorphic fungi; however, additional factors including CO_2_, reactive oxygen species, and steroid hormones are also thought to influence morphogenesis [2]–[5].

The ability of thermally dimorphic fungi to transition between two distinct morphological states in response to environmental stimuli is important for the maintenance of their disparate lifestyles as soil saprobes and mammalian pathogens [6]. Thermally dimorphic fungal pathogens, such as *Histoplasma capsulatum* and *Blastomyces dermatitidis*, grow in the soil in a filamentous or “mold” form that produces vegetative spores known as conidia. Upon inhalation of hyphal fragments or conidia by a human host, and subsequent growth at mammalian body temperature, *H. capsulatum* and *B. dermatitidis* transition into a budding yeast form capable of growth and pathogenesis in mammals. Since thermally dimorphic fungi can persist in mammals after an acute infection is resolved, it is thought that the parasitic host form returns to the soil after the death of infected animal hosts, thus facilitating a transition to the filamentous form and serving to maintain an infectious reservoir [7]–[9]. Maintenance of an environmental reservoir is crucial for the pathogenic lifestyle of thermally dimorphic fungi since these organisms are not directly transmitted between mammalian hosts [10].

Recently, some progress has been made in understanding the molecular mechanisms by which environmental signals stimulate morphological transitions in thermally dimorphic fungi. Comparative gene expression profiling of *H. capsulatum* has revealed that approximately 15% of predicted genes are differentially expressed between the two morphological forms (multicellular filaments and unicellular yeast cells), indicating that a significant fraction of transcripts exhibit yeast-phase or filamentous-phase enrichment expression patterns [11]–[13]. In addition, forward genetic screens have identified regulators of yeast- and filamentous-phase growth in thermally dimorphic fungi, namely, the Ryp (required for yeast-phase growth) master regulators (Ryp1, Ryp2, and Ryp3), the histidine kinase Drk1 (dimorphism-regulating kinase 1), and the GATA-family transcriptional regulator Sre1/SreB [12], [14]–[18]. However, it remains to be elucidated how these regulators sense and integrate environmental signals into phenotypic and genotypic outputs.

One challenge in studying the molecular events involved in the morphogenesis of thermally dimorphic fungi has been establishing a robust and synchronous morphological phase transition *in vitro*. For example, in laboratory cultures of *H. capsulatum*, the conversion between yeast cells and filaments is recapitulated by switching the temperature from 37°C to room temperature (RT) [5], [19]. The switch is bidirectional, so *H. capsulatum* filaments can be switched to yeast cells by shifting the temperature in the opposite direction (RT to 37°C). Under these laboratory conditions, temperature is sufficient to promote morphogenesis; however, the interconversion between yeast cells and filamentous cells in the laboratory is slow and asynchronous, suggesting that other environmental cues that promote morphogenesis are missing from *in vitro* culture. Establishing a robust and synchronous phase transition of thermally dimorphic fungi *in vitro* would allow an easier and more robust examination of the temporal series of events that occurs during morphogenesis, and permit identification of factors important for cellular differentiation.

To identify potential inducers of morphogenesis in the thermally dimorphic fungi, we contemplated developmental pathways in other fungi. The yeast-to-filament transition is well studied in the human commensal fungus *Candida albicans*, and can be induced by a variety of signals [20]. For example, the ubiquitous monosaccharide *N*-acetylglucosamine (GlcNAc) is known to promote filamentation through an unidentified pathway in *C. albicans* [21]. Fungi are likely to encounter environmental sources of GlcNAc since this carbohydrate is a major component of insect exoskeletons and bacterial peptidoglycan. Additionally, the innermost layer of the fungal cell wall is composed of chitin, which is a polymer of β1–4 linked GlcNAc that undergoes turnover during the remodeling of the cell wall that accompanies cell division. Thus, GlcNAc derived from chitin that is released by growing fungal cells is also likely to be available extracellularly. GlcNAc is an interesting monosaccharide to promote cell fate determination in fungi as it has been implicated as a conserved signaling molecule across all kingdoms of life including its ability to promote morphogenesis in bacteria [22] and also function as a dynamic intracellular signaling modification akin to phosphorylation in metazoans (*O*-GlcNAc signaling) [23].

Herein we describe the role of GlcNAc as a potent inducer of the yeast-to-filamentous phase transition at RT in thermally dimorphic fungi. Culturing *H. capsulatum* and *B. dermatitidis* yeast cells in the presence of exogenous GlcNAc promoted a rapid and more synchronous phase transition of yeast cells to filaments. GlcNAc also promoted faster growth of differentiated *H. capsulatum* filaments at RT, indicating that GlcNAc influences both the morphogenesis and growth rate of *H. capsulatum* filaments. In addition to implicating GlcNAc as a critical signal for filamentation, these studies allowed us to examine the temporal regulation of the *H. capsulatum* transcriptome during morphogenesis in a synchronous population of cells. The resulting analysis provided the first view of transcriptional changes of a thermally dimorphic fungus undergoing yeast-to-filament differentiation and revealed candidate genes that may play roles in establishing and maintaining the filamentous growth program. Furthermore, we found that GlcNAc-promoted filamentation of *H. capsulatum* is dependent on two genes (*NGT1* & *NGT2*) that encode putative GlcNAc major facilitator superfamily (MFS) transporters. These proteins have homology to *C. albicans* Ngt1, the only previously characterized eukaryotic GlcNAc transporter [24]. We show that *H. capsulatum* Ngt1 and Ngt2 can each serve as a GlcNAc transporter. Additionally, *NGT1* and *NGT2* were required for efficient yeast-to-filament conversion even in the absence of exogenously added GlcNAc. These data suggest that the ability to sense and respond to endogenous GlcNAc through Ngt transporters could be a critical regulatory step during filamentous growth. Finally, taken together with previous work in *Candida* species, our results indicate that GlcNAc functions as a highly conserved cue to signal morphogenesis in the fungal kingdom.

## Results

### GlcNAc promotes morphogenesis of thermally dimorphic fungi at RT

**Figure 1:**
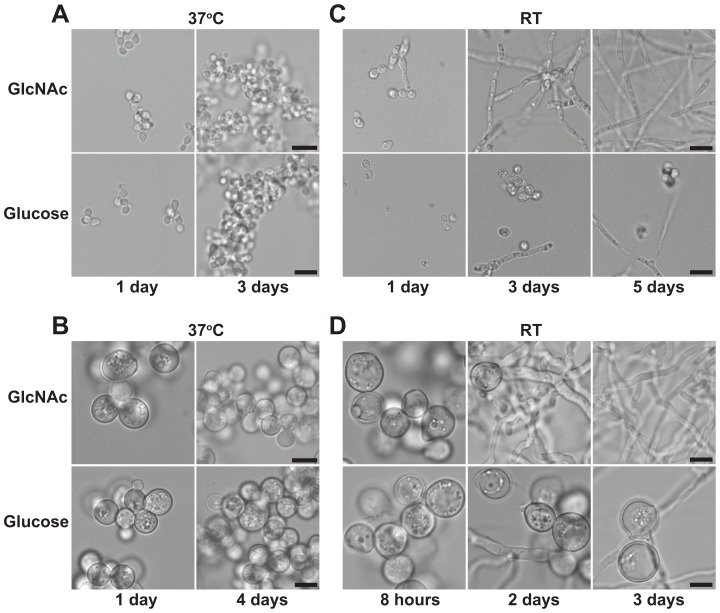
GlcNAc promotes morphogenesis of thermally dimorphic fungi at RT. *H. capsulatum* (A, C) or *B. dermatitidis* (B, D) yeast cells grown at 37°C were inoculated into liquid HMM (“Glucose”) or HMM/100 mM GlcNAc (“GlcNAc”) medium and grown at 37°C to monitor yeast phase growth or transferred to RT to monitor conversion to filaments. Cell morphology was assessed at each indicated timepoint by confocal DIC microscopy on live cells. Scale bar, 10 µm.

**Figure 2:**
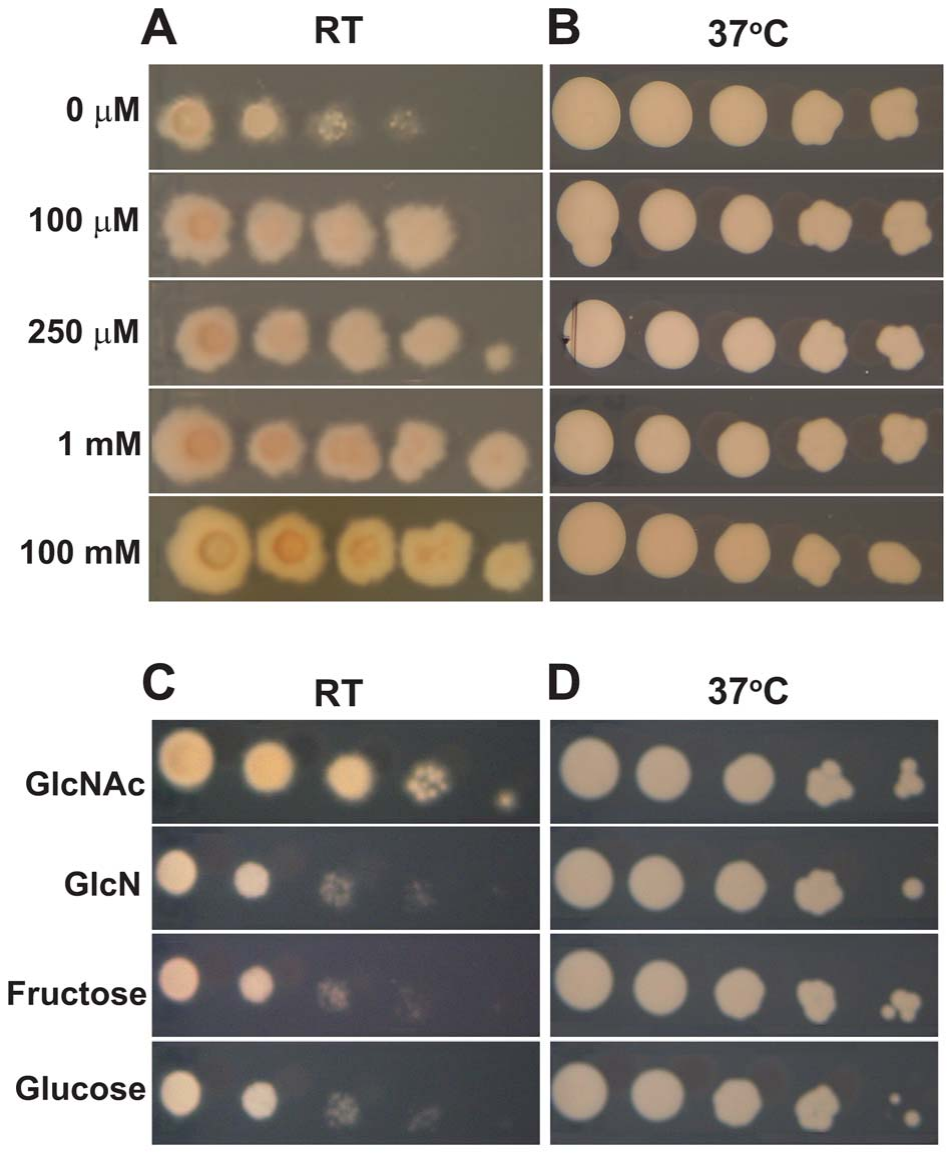
Micromolar concentrations of GlcNAc are sufficient to promote morphogenesis at RT. Ten-fold serial dilutions of *H. capsulatum* yeast cells grown at 37°C were plated onto HMM solid medium supplemented with increasing concentrations of GlcNAc (HMM supplemented with 100 µM–1 mM GlcNAc) or HMM/100 mM GlcNAc medium and transferred to RT to monitor growth in the filamentous form (A) or kept at 37°C to monitor growth in the yeast form (B). Ten-fold serial dilutions of *H. capsulatum* yeast cells grown at 37°C were also plated onto HMM solid medium supplemented with either 1 mM fructose, 1 mM glucosamine (GlcN), or 1 mM GlcNAc and transferred to RT for growth in the filamentous form (C) or kept at 37°C to monitor growth in the yeast form (D). Increased growth of filamentous cells on HMM GlcNAc solid medium at RT is evident by larger colony diameter, fuzzy colony morphology, and increased visible growth at lower dilutions. Representative images are shown.

To determine whether GlcNAc can stimulate morphogenesis of thermally dimorphic fungi, we grew *H. capsulatum* and *B. dermatitidis* yeast cells in liquid culture in the presence or absence of exogenous GlcNAc (HMM/100 mM GlcNAc, see Materials and Methods for complete media descriptions and note that HMM medium contains residual 10 mM glucose from the F12 nutrient supplement even before further supplementation of the sugar source) at 37°C or at RT. GlcNAc did not affect yeast-phase morphology at 37°C (Figure 1 A, B), but it triggered a remarkably rapid transition of yeast cells to filaments at RT (Figure 1 C, D). This robust filamentation was in stark contrast to the usual laboratory transition experiment in glucose medium, where it takes weeks to yield a large, homogenous population of filaments from yeast cells (Figure 1 C, D). To assess the concentration-dependence of GlcNAc-enhanced filamentation, we plated serial dilutions of *H. capsulatum* yeast cells at RT on standard glucose medium containing increasing concentrations of GlcNAc. The enhanced filamentation of *H. capsulatum* at RT in response to GlcNAc occurred at micromolar concentrations, as evidenced by larger colony diameter and fuzzy colony morphology that was in contrast to yeast cells grown in these media conditions at 37°C, which exhibited no morphological changes (Figure 2 A, B). Since these concentrations are too low for GlcNAc to be utilized as the major carbon source, these data suggested that *H. capsulatum* cells may be sensing GlcNAc, or one of its catabolic byproducts, to promote morphological differentiation. To confirm that supplementing cultures with an additional carbon source was not sufficient to promote filamentation, we grew *H. capsulatum* yeast cells in equimolar amounts of glucose or GlcNAc and monitored their conversion to filaments at RT. Cells grown in additional glucose did not show enhanced filamentation at RT in comparison to GlcNAc-grown cells (Figure S1), indicating that simply providing an additional carbon source during aerobic growth is not sufficient to promote morphogenesis of *H. capsulatum*. Furthermore, the ability of GlcNAc to promote filamentous growth in *H. capsulatum* was a unique property of GlcNAc as other carbohydrates, including fructose and the amino sugar glucosamine (GlcN), did not enhance morphogenesis at RT (Figure 2 C, D).

In addition to the ability of GlcNAc to promote a faster yeast-to-filament transition, we also examined the effect of GlcNAc on yeast and filamentous cell growth rates at RT. First, we examined the growth of yeast cells at RT before they converted to filaments in GlcNAc and glucose media. We observed that yeast cells grew at a very slow rate at RT prior to conversion to filaments irrespective of whether GlcNAc was present in the medium (Figure S2 A, B). Thus, GlcNAc does not affect the growth rate of yeast cells at RT before their conversion to filaments. However, monitoring the rate of increase in diameter of filamentous colonies revealed that GlcNAc did augment the growth rate of filaments at RT (Figure S2C) even at low concentrations (10 mM GlcNAc). These data suggested that in addition to promoting a faster yeast-to-filament transition, GlcNAc also enhanced the growth rate of filamentous cells. Overall, these conversion and growth experiments show that GlcNAc promotes a specific, rapid, and synchronous switch of thermally dimorphic fungal yeast cells to filaments at RT and that growth in the filamentous form is stimulated by GlcNAc.

### Synchronous filamentation in the presence of GlcNAc facilitates transcriptional profiling of the morphogenetic transition

**Figure 3:**
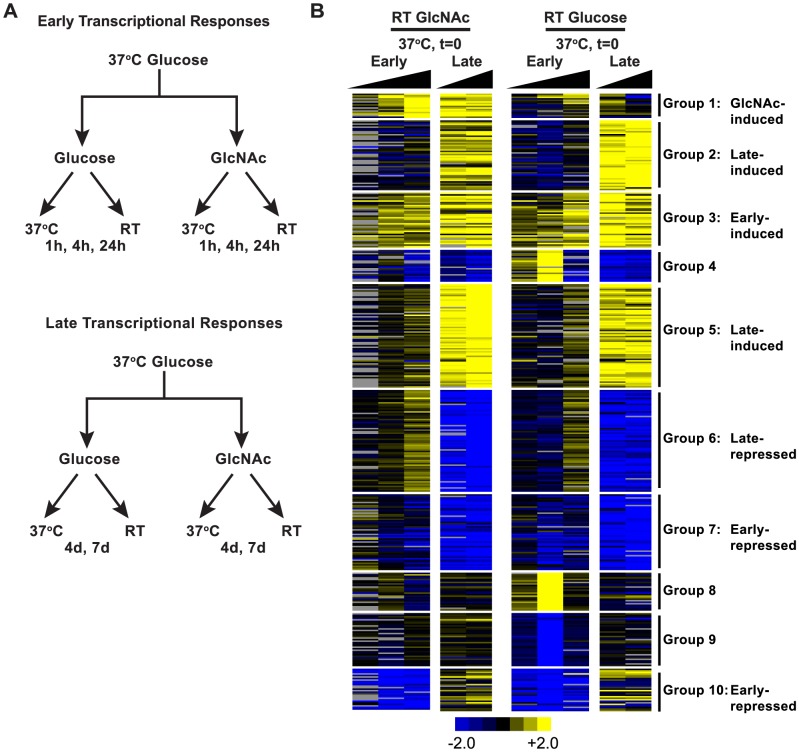
Clustering reveals dynamic control of transcript expression during morphogenesis. (A) Experimental schematic of yeast-to-filament transcriptional profiling. Two independent time courses were performed to catch early (t = 1 h, 4 h, 24 h) and late (4 d, 7 d) transcriptional changes of *H. capsulatum* yeast cells transitioning to filaments in HMM (“Glucose”) or HMM/100 mM GlcNAc (“GlcNAc”) medium at RT. As a point of comparison, identical time courses were performed at 37°C to monitor yeast-phase growth under these experimental conditions. (B) A heatmap of unsupervised k-means clustering of RT timepoints relative to t = 0 (yeast cells, 37°C) was used to identify temporal transcript expression patterns during yeast-to-filament morphogenesis in GlcNAc and glucose media (log_2_-based color scales are shown with grey representing no data available). A CDT file of the k-means cluster is available as Table S1.

The ability of GlcNAc to promote a faster and more synchronous transition of *H. capsulatum* yeast cells to filaments at RT enabled us to examine the transcriptome of *H. capsulatum* cells as they underwent the transition from the yeast form to filaments using whole-transcriptome microarray profiling. Previous transcriptional profiling experiments that defined yeast- and filament-enriched transcripts in *H. capsulatum* have profiled the transcriptomes of fully differentiated yeast cells or filaments grown at 37°C or RT, respectively [11]–[13], which is distinct from deciphering the dynamic changes in transcript expression patterns in cells undergoing a morphological transition. To identify transcripts that are regulated during morphological differentiation as well as to begin to understand how *H. capsulatum* responds to GlcNAc to promote filamentation, we monitored the transcriptome of yeast cells as they began to form filaments at RT in the presence and absence of GlcNAc. We grew yeast cells at 37°C in standard HMM glucose medium to early-log phase (t = 0), resuspended the cells in fresh glucose or GlcNAc medium (HMM/100 mM GlcNAc), and then shifted the yeast cells from 37°C to RT (see Figure 3A) to monitor transcriptional changes that occurred during the yeast-to-filament transition. For technical reasons, two time-courses were performed: the first (1 h, 4 h, 24 h) to capture early transcriptional changes, and the second (4 d, 7 d) to capture later transcriptional changes (see Figure 3A). As a point of comparison, yeast cells were also grown at 37°C in GlcNAc (HMM/100 mM GlcNAc) or glucose medium for the duration of the time-course; these samples allowed us to identify genes that were induced by growth in GlcNAc at both RT and 37°C, independent of temperature. At each timepoint, RNA was harvested and cellular morphology was examined by microscopy. Morphological examination of yeast cells shifted to RT revealed that GlcNAc-grown cells were predominantly filamentous after 4 days of growth at RT while glucose-grown cells remained predominantly yeast-like for the duration of the time-course (Figure S3A). Yeast cells grown at 37°C in either GlcNAc or glucose medium remained as budding yeast for the duration of the time-course (Figure S3B).

Gene expression ratios from cells grown at RT in the presence of GlcNAc or glucose were subjected to k-means clustering, which revealed distinct cases of dynamically controlled transcript expression during morphogenesis. Although a small number of genes were induced or repressed only in glucose or in GlcNAc medium (*e.g.*, Groups 1, 4, 8, and 9, respectively; Figure 3B), the majority of genes showed similar patterns of expression over replicate time courses in the presence of both sugars (Figure 3B). Namely, early repressed, late repressed, early induced, late induced, and GlcNAc induced transcript expression patterns emerged as yeast cells transitioned to filaments in GlcNAc and glucose media (Figure 3B). Overall, these data highlighted the complex reprogramming that yeast cells employ during cellular differentiation.

**Figure 4:**
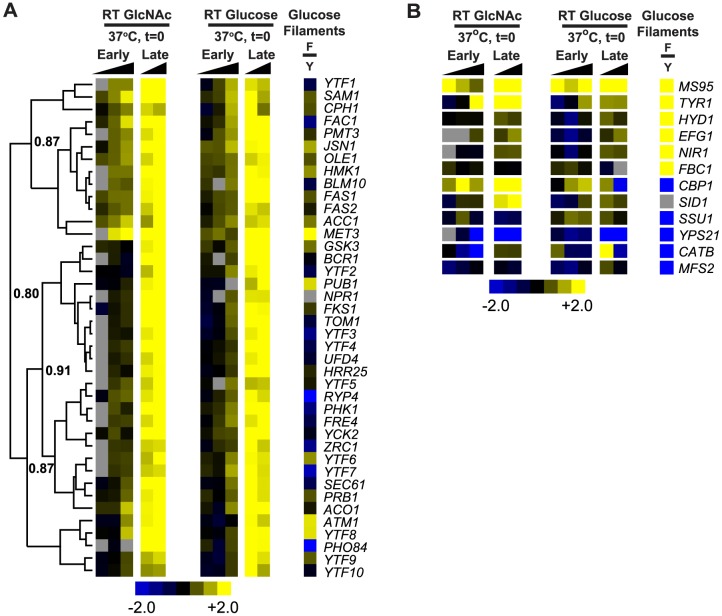
Temporal transcriptional profile of yeast cells converting to filaments. (A) A heatmap highlighting an unsupervised hierarchical cluster of transcripts upregulated during the yeast-to-filament morphogenesis time course at RT as compared to t = 0 (yeast cells, 37°C). Uncentered Pearson correlation coefficients are indicated at each major distance node. (B) A heatmap representing the temporal expression patterns of select FPS and YPS transcripts during the yeast-to-filament transition at RT as compared to t = 0 (yeast cells, 37°C). Log_2_-based color scales are shown for all heatmaps. For comparison and as a point of reference, heatmaps showing the expression pattern for each transcript in fully differentiated glucose-grown filaments (F) as compared to mid-log phase glucose-grown yeast cells (Y) are shown (“Glucose Filaments”; yellow, filament-enriched and blue, yeast-enriched; see Materials and Methods for growth conditions). Grey indicates no data are available. See Table S2 for gene identities and annotation of depicted transcripts. Genes that were difficult to name based on homology were named YTF for Yeast-To-Filament enriched transcript. A CDT file of the hierarchical clustering of the data is available as Table S3.

Closer examination of the identities of temporally regulated transcripts during morphogenesis uncovered genes that may play a role in establishing the filamentous growth program. Factors involved in fatty acid biosynthesis (*FAS1*, *FAS2*, *ACC1*, & *OLE1*) [25], which could serve to alter plasma membrane fluidity in response to a change in temperature or cell wall structure, were upregulated in glucose- and GlcNAc-grown yeast cells transitioning to filaments (Figure 4A). We also found genes upregulated during cellular differentiation that could play a role in signal transduction including *HMK1*, which is predicted to encode a mitogen activated protein kinase, *RYP4* and *CPH1*, which are predicted to encode Zn_2_C_6_ and C_2_H_2_ transcription factors, respectively, and *PHK1*, a predicted two component sensor kinase. Notably, the *S. cerevisiae* homolog of *HMK1* (named *KSS1*) is involved in signal transduction pathways that control filamentous growth [26]. Interestingly, many of the transcripts depicted here (Figure 4A) that were upregulated during morphogenesis are not strongly enriched in fully differentiated filaments, further suggesting that these transcripts may play a role in establishing, but not maintaining, the filamentous growth program.

To put the transcriptional expression patterns observed during morphogenesis in context with what is already known about transcript levels in fully differentiated filaments, we examined the expression patterns of canonical filament-phase specific (FPS) transcripts (*MS95*, *TYR1*, *HYD1*, *EFG1*, *NIR1*, and *FBC1* [11], [12]) during morphogenesis. Transcript levels of *MS95* were upregulated immediately (t = 1 h) upon shifting yeast cells to RT in both GlcNAc and glucose growth conditions, suggesting that *MS95* is responsive to the decrease in temperature, either at an extremely early point in the yeast-to-filament transition or even before that developmental program has initiated (Figure 4B). *MS95* is homologous to the *C. albicans DDR48* gene, which is a stress-response gene that is important for filamentous growth in *C. albicans* [27]. In contrast to *MS95*, upregulation of the predicted tyrosinase-encoding *TYR1* transcript in yeast cells transitioning to filaments was not observed at early timepoints after temperature shift (1 h or 4 h; Figure 4B), signifying that *TYR1* may be induced once the filamentous growth program has initiated, and is not directly regulated by temperature. To further examine the transcriptional expression kinetics of *TYR1* during filamentation, we introduced a GFP reporter construct in which expression of GFP was controlled by approximately 1 kilobase (kb) of the *TYR1* promoter (P*_TYR1_* - GFP) into *H. capsulatum* and monitored levels of GFP by confocal microscopy as yeast cells transitioned to filaments at RT in glucose or GlcNAc medium. In accordance with our microarray data, yeast cells converting to filaments in GlcNAc medium exhibited earlier P*_TYR1_* - GFP expression (robust GFP signal detected by 36 h) than glucose-grown cells (Figure S4). Furthermore, P*_TYR1_* - GFP expression was not detected in yeast cells grown at 37°C (data not shown), nor was P*_TYR1_* - GFP expression immediately observed upon the transition of yeast cells to RT (t = 12 h or 20 h, Figure S4). Together, these data confirm the enhanced rate of filamentation in GlcNAc medium, and indicate that the *TYR1* transcript is turned on during the filamentous growth program and not directly in response to changes in temperature.

The FPS *HYD1*, *EFG1*, *NIR1*, and *FBC1* transcripts, in contrast to *MS95* and *TYR1*, were not robustly upregulated in either glucose- or GlcNAc-grown yeast cells transitioning to filaments despite being highly enriched in fully differentiated glucose-grown filaments (Figure 4B). Thus, *HYD1*, *EFG1*, *NIR1*, and *FBC1* transcripts may not be involved in establishment of the filamentous state, and may instead play a role in maintenance of this cell type. We also examined the temporal expression patterns of canonical yeast-phase specific (YPS) transcripts in our transcriptional time-course with the expectation that we would see expression of YPS transcripts such as *CBP1*, *SID1*, *SSU1*, *YPS21*, *CATB*, and *MFS2* [11], [28]–[30] downregulated as the yeast cells converted to filaments. *SSU1*, *YPS21*, *CATB*, and *MFS2* transcripts behaved as we expected, being unchanged or downregulated in expression upon shifting yeast cells to RT (Figure 4B). *CBP1* and *SID1*, however, were unexpectedly more upregulated in GlcNAc- versus glucose-grown yeast cells transitioning to filaments (Figure 4B; upregulation of *CBP1* was confirmed by qRT-PCR (data not shown)). *CBP1*, a gene of unknown molecular function, and *SID1*, a monooxygenase involved in siderophore biosynthesis, are required for the virulence of *H. capsulatum* yeast cells [31]–[33]. The upregulation of *CBP1* and *SID1* transcripts in GlcNAc-grown filaments may reflect a previously unappreciated function for these transcripts in the biology of filamentous cells. Additionally, since GlcNAc-stimulated filaments also grow more rapidly than glucose-grown filaments (see Figure S2C), these data could reflect a correlation between increased growth rate of cells and increased expression of *CBP1* and *SID1*. The unexpected expression patterns of these previously identified phase-specific genes highlight the importance of investigating the temporal regulation of transcripts during morphogenesis.

### GlcNAc induces a unique transcriptional program

**Figure 5:**
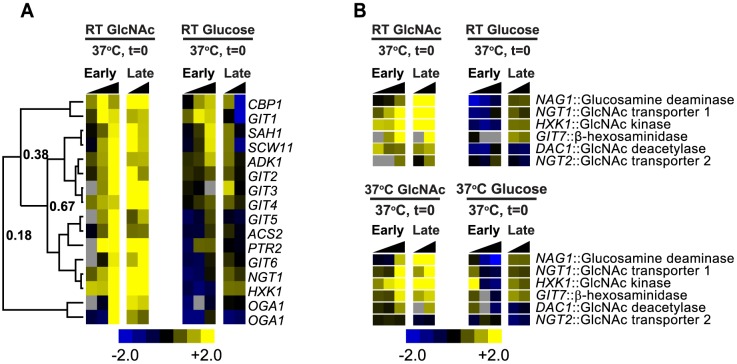
GlcNAc induces a unique transcriptional program. (A) A heatmap highlighting an unsupervised cluster of transcripts upregulated more robustly in GlcNAc- versus glucose-grown cells during the yeast-to-filament morphogenesis time course at RT as compared to t = 0 (yeast cells, 37°C). Two different oligos are present for *OGA1* on our array design. Uncentered Pearson correlation coefficients are indicated at each major distance node. (B) A heatmap demonstrating the expression profiles of putative GlcNAc utilization genes during the yeast-to-filament morphogenesis time course at RT as compared to t = 0 (yeast cells, 37°C). Log_2_-based color scales are shown for all heatmaps. Grey indicates no data are available. See Table S4 for gene identities and annotation of depicted transcripts. A CDT file of the hierarchical clustering of the data is available as Table S3. Genes that were difficult to name based on homology were named GIT for GlcNAc-Induced Transcript.

In addition to the aforementioned transcripts temporally regulated in both GlcNAc- and glucose-grown yeast cells transitioning to filaments, we also identified a class of transcripts robustly induced only in GlcNAc medium (Group 1, Figure 3B). This group of genes was of interest, as it represents a means to understand the mechanism of GlcNAc-promoted filamentation. Further analysis of GlcNAc-induced genes using hierarchical clustering identified a subset of genes that is robustly induced in GlcNAc but not glucose medium at RT (Figure 5A). Whereas many of the GlcNAc-induced transcripts are of unknown function, we noted that *OGA1* was particularly intriguing because it could play a signaling role in GlcNAc-promoted filamentation. *OGA1* encodes a putative *O*-GlcNAcase with homology to the human OGA enzyme that cleaves the *O*-GlcNAc signaling modification from serine and threonine residues [23]. This modification is involved in a variety of signaling processes in metazoan cells [34], but has not been functionally investigated in fungal cells. *OGA1* has not previously been identified as a filament-enriched transcript [12] and is induced during the rapid and synchronous transition to filaments in GlcNAc medium. Therefore, we hypothesize that it could play a role in the robust morphologic transition to filaments that occurs in GlcNAc-treated cells. Of note, the *H. capsulatum* homologs of *GIG1* and the galactose catabolic genes were not found to be induced by GlcNAc in *H. capsulatum* as they are in *C. albicans* [35], indicating that their regulation may not be significant for GlcNAc-promoted filamentation of thermally dimorphic fungi.

GlcNAc also induced the expression of putative GlcNAc utilization genes in *H. capsulatum*, including a GlcNAc transporter (*NGT1*), GlcNAc hexokinase (*HXK1*), GlcNAc-6-phosphate deacetylase (*DAC1*), and GlcN-6-phosphate deaminase/isomerase (*NAG1*; Figure 5 A, B and see Figure S5). These genes were induced in GlcNAc at both RT and 37°C independent of cellular morphology (Figure 5B). *H. capsulatum* Ngt1, Hxk1, Dac1, and Nag1 are the best BLASTP hits to the functionally characterized *C. albicans* GlcNAc utilization machinery (see Materials and Methods) [24], [36], [37]. Analysis of the genomic positions of *H. capsulatum NGT1*, *HXK1*, *DAC1*, and *NAG1* genes showed that they are clustered in a 75 kb region in the *H. capsulatum* genome similarly to the *C. albicans NAG1*, *DAC1*, and *HXK1* GlcNAc catabolic genomic cluster [38] (see Figure S6 and Table S6). We identified a fifth putative GlcNAc utilization gene (HISTO_ZL.Contig1131.fgenesh_plus.101.final_new) by its location in the *H. capsulatum* GlcNAc utilization genomic cluster (Figure S6 and Table S6) and its transcriptional induction by GlcNAc (Figure 5B). This gene, which we named *GIT7* (GlcNAc-Induced Transcript 7), is predicted to encode a β-hexosaminidase domain (Panther Accession: PTHR30480). Despite the conservation of Git7 across many fungal species, including *C. albicans*, *Aspergillus fumigatus*, and *B. dermatitidis*, the biological function of Git7 in GlcNAc utilization is unknown. Together, these data suggest that the *H. capsulatum* genome encodes and transcribes all of the genes known to be necessary for GlcNAc catabolism as well as an additional uncharacterized gene, *GIT7*, which could be involved in GlcNAc utilization.

### Evolutionary conservation of multiple GlcNAc transporters across filamentous fungi

**Figure 6:**
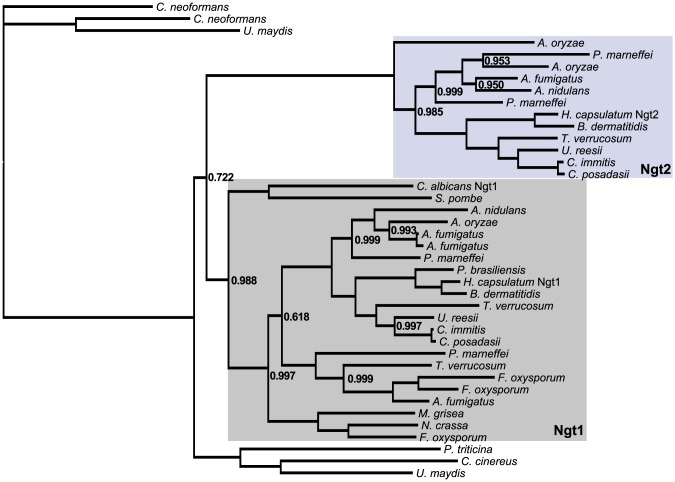
Evolutionary conservation of GlcNAc transporters in fungi. An unrooted phylogenetic tree generated by Bayesian analysis demonstrates evolutionary conservation of multiple GlcNAc transporters in some fungal species. *H. capsulatum*, along with *A. fumigatus*, *A. nidulans*, *A. oryzae*, *B. dermatitidis*, *C. immitis*, *C. posadasii*, *P. marneffei*, *T. verrucosum*, and *U. reesii* form a sister clade of genes orthologous to Ngt1, which we have named Ngt2. Probability values that are less than 1.0 for one million iterations are shown. Protein accession numbers are given in Table S5.

After identifying *H. capsulatum* Ngt1, we also noticed a second previously uncharacterized gene (HISTO_ER.Contig17.eannot.1311.final_new) with strong sequence homology to the *C. albicans* Ngt1 transporter by BLASTP analysis (E = 8.2×10^−90^). Since this gene is predicted to encode a second Ngt-like transporter in *H. capsulatum*, we named it Ngt2. Similarly to the *C. albicans* Ngt1 [24], Ngt2 is predicted to be a MFS transporter (Interpro Accession: IPR011701) with 12 transmembrane-spanning regions. However, in contrast to *NGT1*, *NGT2* mRNA is not robustly induced by GlcNAc (Figure 5B) nor is *NGT2* clustered in the genome near other *H. capsulatum* GlcNAc utilization genes (see Figure S6 and Table S6). The presence of two putative GlcNAc transporters in the *H. capsulatum* genome (*NGT1* & *NGT2*) is surprising as *C. albicans* requires only one transporter, Ngt1, to transport GlcNAc across its cell membrane [24]. To determine the prevalence of multiple GlcNAc transporters across fungal species, we assessed the phylogenetic conservation of *H. capsulatum* Ngt1 and Ngt2. We used Bayesian analysis to build a phylogenetic tree with aligned BLASTP homologs to *H. capsulatum* Ngt1 and Ngt2 (E≤1×10^−5^) from 20 Ascomycetes and Basidiomycetes species with sequenced genomes (Figure S7). From this analysis, an Ngt1/Ngt2 clade was identified and the phylogenetic model was simplified and then rebuilt to include only species with Ngt1 or Ngt2 orthologs (Figure 6 and Table S5). Ngt1 orthologs were found throughout the Ascomycetes; notably, however, the model yeast *S. cerevisiae* lacks a homolog to Ngt1 and experimentally has been shown to be unable to efficiently transport GlcNAc across its cell membrane [24]. Conversely, the presence of Ngt2 in fungi is more restricted than the occurrence of Ngt1, with Ngt2 orthologs found only in the Onygenales (*H. capsulatum*, *B. dermatitidis*, *Coccidioides* spp., *Trichophyton verrucosum*, and *Uncinocarpus reesii*) and Eurotiales (*Penicillium marneffei* and *Aspergillus* spp.) orders of Ascomycetes (Figure 6). Many of the species that have multiple identifiable GlcNAc transporters (*i.e.*, Ngt1 and Ngt2) are human fungal pathogens (*H. capsulatum*, *B. dermatitidis*, *Coccidioides* spp., *T. verrucosum*, *A. fumigatus*, and *P. marneffei*), which is interesting given that the majority of characterized Ascomycetes are plant pathogens or plant saprobes [39].

### *NGT1* and *NGT2* transport GlcNAc to allow for efficient GlcNAc catabolism

**Figure 7:**
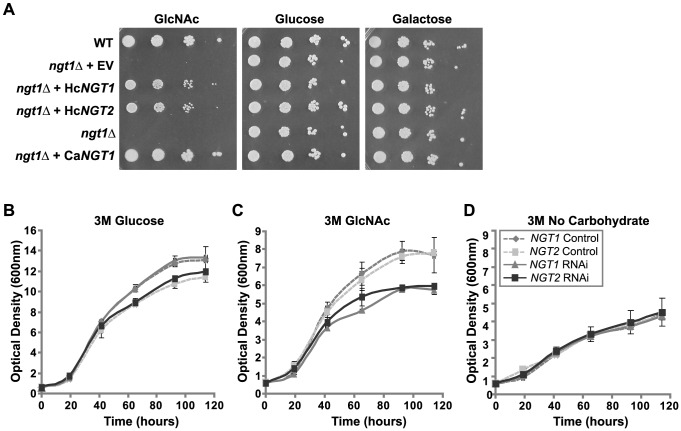
*H. capsulatum* Ngt1 and Ngt2 facilitate GlcNAc transport. (A) Expression of *H. capsulatum NGT1* or *NGT2* restores growth of the *C. albicans ngt1*Δ in GlcNAc media. *H. capsulatum NGT1* (Hc*NGT1*), *H. capsulatum NGT2* (Hc*NGT2*), *C. albicans NGT1* (Ca*NGT1*), or empty vector (EV) were introduced into *C. albicans ngt1*Δ yeast cells (*ngt1*Δ). Transformants were examined for growth on solid medium containing 50 mM glucose, galactose, or GlcNAc as the sole carbon source by plating serial dilutions of each strain and comparing growth to wild-type *C. albicans* (WT). Representative images are shown. (B, C, D) *H. capsulatum NGT1* and *NGT2* are necessary for growth of *H. capsulatum* in medium where GlcNAc is the only carbohydrate source. Vector control, *NGT1* RNAi, and *NGT2* RNAi strains were starved overnight to deplete available carbohydrate sources and then inoculated into 3M minimal medium containing (B) glucose, (C) GlcNAc, or (D) no carbohydrate. At each indicated timepoint, growth was evaluated by measuring the optical density at 600 nm. The standard deviation of mean OD_600_ values from three independent RNAi and vector control clones for each knockdown construct are shown.

To investigate whether *H. capsulatum* Ngt1 and Ngt2 are capable of transporting GlcNAc, we expressed *H. capsulatum NGT1* and *NGT2* in the *C. albicans ngt1*Δ strain, which is unable to grow in medium where GlcNAc is the sole carbon source [24]. We overexpressed either *H. capsulatum NGT1* or *NGT2* in the *C. albicans ngt1*Δ mutant and examined the ability of these strains to grow in GlcNAc medium as compared to vector control strains. Expression of either *H. capsulatum NGT1* or *NGT2* in the *C. albicans ngt1*Δ mutant conferred growth on GlcNAc medium (Figure 7A), indicating that both *H. capsulatum* Ngt1 and Ngt2 can mediate GlcNAc transport independently. As expected, complementation of the *C. albicans ngt1*Δ mutant with either *H. capsulatum NGT1* or *NGT2* did not affect growth on other carbon sources including glucose and galactose (Figure 7A).

To probe whether *NGT1* and *NGT2* function in GlcNAc transport and utilization in *H. capsulatum*, we attempted to disrupt each gene, but were unsuccessful since targeted gene disruption in *H. capsulatum* is highly inefficient. Thus we used RNA interference (RNAi) to deplete levels of *NGT1* and *NGT2* transcripts. We confirmed knockdown of *NGT1* and *NGT2* transcripts by qRT-PCR and noted that wild-type levels of *NGT1* mRNA were dependent on *NGT2* whereas depleting levels of *NGT1* had little effect on *NGT2* transcript levels (Figure S8 A, B). Nucleotide regions that were unique to either *NGT1* or *NGT2* were chosen for targeting by RNAi to minimize the possibility of cross-silencing of *NGT1* by the *NGT2* RNAi construct, or *vice versa*. With *NGT1* and *NGT2* RNAi strains in hand, we evaluated the ability of these strains to grow in medium where GlcNAc was the only carbohydrate source. We grew vector control, *NGT1*, and *NGT2* RNAi strains in minimal medium (3M) using either GlcNAc or glucose as the only carbohydrate source. As predicted, *NGT1* and *NGT2* RNAi strains exhibited no growth defects in glucose medium, indicating that *NGT1* and *NGT2* do not affect overall fitness of *H. capsulatum* at 37°C (Figure 7B). However, in medium where GlcNAc was the only carbohydrate source, both *NGT1* and *NGT2* RNAi strains were unable to achieve the same growth density as control cells (Figure 7C). In contrast, the baseline level of growth exhibited by *H. capsulatum* in minimal medium containing no exogenous sugar source was indistinguishable for *NGT1* and *NGT2* RNAi strains and controls (the carbon source for these cells is presumed to be the proline and cystine present in 3M minimal media; Figure 7D).

**Figure 8:**
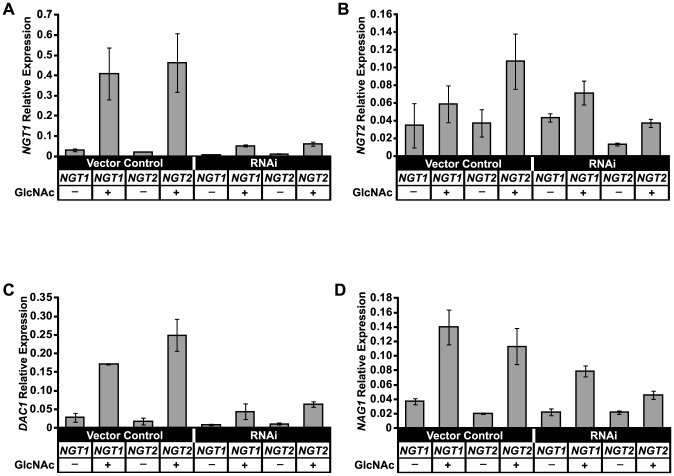
*NGT1* and *NGT2* are required for maximal induction of GlcNAc utilization genes. *NGT1* and *NGT2* transcript levels were depleted by RNAi in *H. capsulatum* and qRT-PCR was used to determine levels of (A) *NGT1* (B) *NGT2* (C) *DAC1* and (D) *NAG1* transcripts in each RNAi knockdown strain as compared to vector control cells in the absence (HMM glucose) or presence of GlcNAc (HMM/100 mM GlcNAc). Transcript levels were normalized to *ACT1*. The standard deviation of mean expression values from three independent RNAi and vector control clones is shown; p-values ≤0.02 for the comparisons of vector control versus *NGT* RNAi during GlcNAc induction of *NGT1*, *DAC1*, and *NAG1* transcripts.

Given that *H. capsulatum* cells respond to GlcNAc by upregulating GlcNAc utilization genes (Figure 5B), we predicted that *NGT1* and *NGT2* RNAi strains would be defective in the upregulation of *NAG1*, *DAC1*, and *NGT1* transcripts in response to GlcNAc. We first examined the responsiveness of *NGT1* and *NGT2* expression levels to GlcNAc. Whereas *NGT1* is induced approximately 13 fold by GlcNAc in control cells, GlcNAc induction of *NGT1* is reduced in strains that target either *NGT1* or *NGT2* by RNAi (Figure 8A). In contrast, as observed by microarray and qRT-PCR, the expression level of *NGT2* was only minimally affected by GlcNAc (Figure 5B and 8B), and was not dependent on *NGT1* (Figure 8B). Maximal induction of *NAG1* and *DAC1* transcripts by GlcNAc was abolished in *NGT1* and *NGT2* RNAi strains (Figure 8 C, D), suggesting that Ngt1 and Ngt2 play a role in GlcNAc catabolism and utilization in *H. capsulatum*. Interestingly, we also noticed that *NGT1* RNAi strains exhibited a slight defect in upregulation of the GlcNAc catabolic genes *NAG1* and *DAC1* even in the absence of adding exogenous GlcNAc to the medium (Figure S9 A, B). These data support the idea that cells might scavenge endogenous GlcNAc that becomes available as cells divide (*i.e.*, from cell wall turnover during growth in glucose as the major carbon source) and indicate that *NGT1* is required for this process. In sum, these data indicated that Ngt1 and Ngt2 most likely contribute to GlcNAc catabolism in *H. capsulatum* by mediating GlcNAc transport.

In addition to examining the role of *NGT1* and *NGT2* in regulating genes involved in GlcNAc catabolism, we also examined whether they regulate the expression of GlcNAc-induced transcripts (Figure 5A) that do not have a predicted role in GlcNAc catabolism. We examined the upregulation of *PTR2* and *OGA1* transcripts in response to GlcNAc in the *NGT1* and *NGT2* RNAi strains by qRT-PCR. We observed that *OGA1* expression was dependent on *NGT1* and *NGT2* while *PTR2* expression was dependent only on wild-type levels of *NGT2* (Figure S10). Similarly to the GlcNAc catabolic genes *NAG1* and *DAC1*, *OGA1* expression was dependent on *NGT1* even in the absence of exogenous GlcNAc (Figure S9 C, D), providing more evidence that Ngt1 influences the expression of GlcNAc-induced transcripts even when glucose is the major carbon source.

### *NGT1* and *NGT2* are required for efficient morphogenesis of *H. capsulatum*

**Figure 9:**
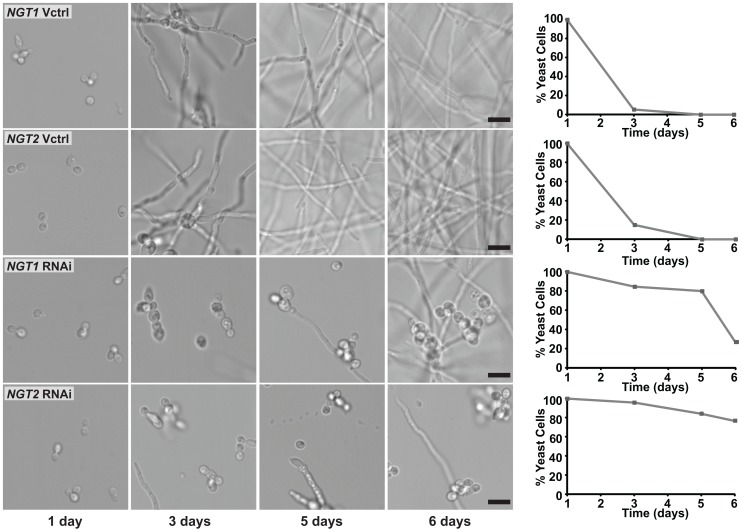
GlcNAc transporters mediate GlcNAc-promoted *H. capsulatum* morphogenesis. *H. capsulatum* yeast cells grown at 37°C were inoculated into liquid HMM medium supplemented with 10 mM GlcNAc and transferred to RT to monitor conversion to filaments. Cell morphology was assessed at each indicated timepoint by live-cell confocal DIC microscopy and scored to assess the percentage of yeast cells remaining at each timepoint. Scale bar, 10 µm. Representative images are shown.

In *C. albicans*, GlcNAc-mediated filamentation is dependent on Ngt1, but independent of Hxk1 [24], [40]; Hxk1 is required for catabolism of GlcNAc as a carbon source and utilization of GlcNAc in glycan biosynthesis (see Figure S5). These data indicate that GlcNAc is likely being recognized intracellularly by *C. albicans* as opposed to being catabolized or utilized in glycan biosynthesis to promote filamentous growth. Furthermore, in *H. capsulatum* we found that Ngt1 and Ngt2 were necessary for the induction of transcripts that were upregulated during the GlcNAc-promoted yeast-to-filament transition. Thus, to investigate the role of *H. capsulatum* Ngt1 and Ngt2 in morphogenesis, we first confirmed that expression of *H. capsulatum NGT1* or *NGT2* in the *C. albicans ngt1*Δ strain restored filamentation in response to GlcNAc (Figure S11). Next, we evaluated whether the *H. capsulatum NGT1* and *NGT2* RNAi yeast cells were defective in GlcNAc-induced filamentation. Vector control, *NGT1*, and *NGT2* RNAi yeast cells grown at 37°C were inoculated into either HMM glucose medium or HMM glucose medium supplemented with 10 mM GlcNAc and switched to RT to monitor their conversion to filaments over time by live-cell imaging. Depletion of *NGT1* or *NGT2* transcripts resulted in a dramatically slower conversion of yeast cells to filaments in GlcNAc medium as compared to vector control strains (Figure 9), indicating that *NGT1* and *NGT2* mediate GlcNAc-promoted filamentation. To investigate whether *NGT*-mediated GlcNAc filamentation was dependent on the catabolism or utilization of GlcNAc by *H. capsulatum* cells, we depleted transcript levels of the GlcNAc kinase, *HXK1*, using RNAi (Figure S8C). Depletion of *HXK1* severely reduced the ability of *H. capsulatum* cells to grow in minimal medium (3M) containing GlcNAc as the only carbohydrate source (Figure S12A), demonstrating that *HXK1* functions as a GlcNAc kinase during catabolism. Next, we evaluated the ability of *HXK1* RNAi yeast cells to filament in response to GlcNAc and noted no defect in the ability of *HXK1* RNAi yeast cells to transition to filaments in response to GlcNAc as compared to vector control cells (Figure S12 B, C). Thus, in *H. capsulatum NGT*-mediated filamentation in response to GlcNAc does not require GlcNAc catabolism or utilization.

**Figure 10:**
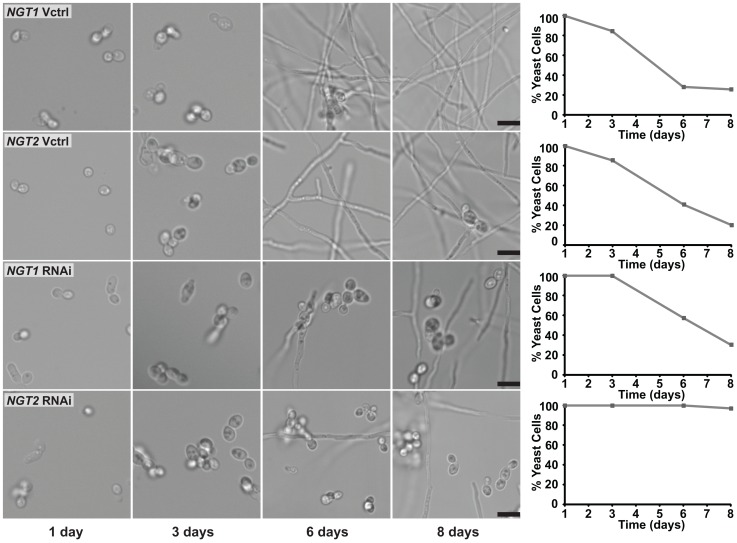
GlcNAc transporters are necessary for efficient *H. capsulatum* morphogenesis in glucose medium. *H. capsulatum* yeast cells grown at 37°C were inoculated into liquid HMM medium (glucose only) and transferred to RT to monitor conversion to filaments. Cell morphology was assessed at each indicated timepoint by live-cell confocal DIC microscopy and scored to assess the percentage of yeast cells remaining at each timepoint. Scale bar, 10 µm. Representative images are shown.

Unexpectedly, *NGT1* and *NGT2* RNAi yeast cells also exhibited a defect in the ability to convert to filamentous cells at RT in glucose medium (Figure 10), indicating that Ngt1 and Ngt2 play a general role in mediating the yeast-to-filament transition at RT in *H. capsulatum*. We hypothesized that the GlcNAc polymer chitin found in fungal cell walls could be a source of endogenous GlcNAc for *H. capsulatum* cells; thus, we examined whether chitin could stimulate morphogenesis similarly to GlcNAc. Indeed, *H. capsulatum* yeast cells grown in the presence of chitin more robustly converted to filaments at RT (Figure S13), suggesting a potential endogenous source of GlcNAc that *H. capsulatum* cells could be monitoring. Taken together, our experiments indicate that exogenously added GlcNAc stimulates a morphogenetic pathway in *H. capsulatum* that facilitates temperature-dependent filamentous growth in a Ngt1- and Ngt2-dependent manner. Additionally, our data reveal that Ngt1 and Ngt2 are required for efficient filamentation in the absence of GlcNAc supplementation, likely due to the role of endogenous GlcNAc in the regulation of morphogenesis.

## Discussion

Here we demonstrated that the ubiquitous amino sugar GlcNAc robustly promotes morphogenesis of the thermally dimorphic fungal pathogens, *H. capsulatum* and *B. dermatitidis*. Historically, temperature has been thought of as the only signal necessary to induce morphogenesis of thermally dimorphic fungi; however, the discovery that exogenous GlcNAc represents a secondary signal important for efficient yeast-to-filament conversion indicates that combinatorial signals are integrated by thermally dimorphic fungi to cue morphogenesis. In addition to its role in promoting morphogenesis, GlcNAc also stimulated faster growth of differentiated *H. capsulatum* filamentous cells at RT. This was surprising as GlcNAc does not appear to be the optimal carbon source for *H. capsulatum* (glucose is a more efficiently utilized carbon source by *H. capsulatum* yeast cells *in vitro*), and suggests that GlcNAc stimulates filamentous growth by a mechanism independent of metabolic flux. In trying to understand the mechanism of GlcNAc-promoted filamentation, we focused on genes that were transcriptionally co-regulated in response to GlcNAc. Interestingly, this analysis led to the observation that GlcNAc-promoted morphogenesis of *H. capsulatum* is dependent on two GlcNAc transporters, Ngt1 and Ngt2. Furthermore, Ngt1 and Ngt2 were necessary for efficient yeast-to-filament morphogenesis even in the absence of exogenous GlcNAc, suggesting that Ngt1 and Ngt2 may function as part of a general autoregulatory mechanism in *H. capsulatum*, presumably dependent on endogenous GlcNAc (*i.e.*, GlcNAc that could be turned over from the remodeling of chitin in the cell wall that accompanies cell division), that serves to control multicellular filamentous growth.

The kinetics and synchrony of the temperature-induced morphologic switch were greatly enhanced by GlcNAc supplementation, which allowed more robust profiling of the yeast-to-filament transition. Classifying transcript expression patterns based on their temporal regulation during the yeast-to-filament transition of *H. capsulatum* yielded distinct categories of regulated transcripts. Surprisingly, we noticed similar expression patterns for regulated transcripts in *H. capsulatum* yeast cells transitioning to filaments at RT in GlcNAc and glucose medium in spite of their disparate cellular morphologies. This could indicate that many of the transcriptional changes that occur during morphogenesis are induced by temperature as opposed to after initiation of the morphologic program. Alternatively, the transcriptional changes we described may not be sufficient to establish morphogenesis, and key transcriptional or post-transcriptional controls of morphogenesis could remain to be identified. Some genes that were induced during the yeast-to-filament transition, but not during static, long-term growth of the filamentous form, included genes that are known to influence filamentation in other, better characterized fungi, making these genes attractive candidates for conserved regulators of filamentous growth across dimorphic fungi. Of note, the transcription factor Cph1 (alias Ste12 in *S. cerevisiae*), which we found upregulated during the yeast-to-filament transition in *H. capsulatum*, is required for filamentous growth in both *C. albicans* [41] and *S. cerevisiae* [42]. Intriguingly, we also noted the upregulation of a mitogen-activated protein kinase, *HMK1*, during *H. capsulatum* morphogenesis. Hmk1 homologs (Kss1 and Cek1 in *S. cerevisiae* and *C. albicans*, respectively) function directly upstream of the Cph1 and Ste12 transcription factors in the pathways controlling filamentous growth in *C. albicans* [43] and *S. cerevisiae* [26]. In addition to implicating genes in the regulation of morphogenesis in thermally dimorphic fungi, our temporal transcriptome analysis revealed surprisingly dynamic expression patterns for genes that were originally characterized as showing differential expression in static samples of either yeast or filaments. We hypothesize that this reflects unanticipated roles for these transcripts in other aspects of fungal cell biology, highlighting the need to profile expression patterns of dimorphic fungal transcripts over a variety of cellular conditions and timepoints to fully characterize phase-specific changes in gene expression.

From our transcriptional profiling data, we chose to focus on the class of genes transcriptionally co-regulated by GlcNAc to begin to understand the mechanism underlying GlcNAc-stimulated morphogenesis. Our work indicated that the *H. capsulatum* GlcNAc transporters, Ngt1 and Ngt2, are required for efficient GlcNAc-mediated filamentation. Whether Ngt1 and Ngt2 ultimately mediate GlcNAc-promoted filamentation by directly sensing GlcNAc or controlling GlcNAc transport for intracellular sensing is unclear. We found that micromolar amounts of GlcNAc were sufficient to promote filamentous growth in *H. capsulatum* even in the presence of 100 mM glucose, suggesting that GlcNAc does not need to be the main carbon source to exert its effects. This also indicates that thermally dimorphic fungi are exquisitely sensitive to levels of extracellular GlcNAc such that low levels of this amino sugar are sufficient to promote robust filamentous growth. Furthermore, we found that knocking down *HXK1*, the GlcNAc kinase which phosphorylates intracellular GlcNAc as the first enzymatic step necessary for utilizing GlcNAc in glycan biosynthesis or catabolism (see Figure S5), does not alter the ability of *H. capsulatum* to filament in response to GlcNAc. Thus, we favor the idea that thermally dimorphic fungi are remarkably sensitive to levels of GlcNAc, such that low levels of this amino sugar are sufficient to promote robust filamentous growth in conjunction with the appropriate temperature signal. External GlcNAc might be sensed by Ngt1/Ngt2, or alternatively, GlcNAc could be transported into the cell and sensed by an intracellular mechanism. To date, no GlcNAc sensing mechanism has been identified in fungi and thus, how GlcNAc fits into the complex regulatory networks that control fungal morphogenesis is unknown.

The identification of GlcNAc-induced transcripts may provide clues into a mechanism for GlcNAc-promoted filamentation. Most notably, we identified *OGA1* as a GlcNAc-induced transcript in *H. capsulatum* and demonstrated that Ngt1 or Ngt2 was necessary for its upregulation in response to GlcNAc. *OGA1* shares homology with the metazoan *O*-GlcNAcase (OGA) enzyme that functions as a regulator of the dynamic intracellular metazoan signaling modification termed *O*-GlcNAcylation [34]. In metazoans, *O*-GlcNAcylation is a ubiquitous post-translational modification, akin to phosphorylation, that regulates basic cellular processes such as cellular development, transcription, protein turnover, and the cell cycle [34]. It is unknown whether fungi modify proteins with *O*-GlcNAc, let alone utilize *O*-GlcNAc as a signaling modification. Future work will examine whether the *O*-GlcNAc modification exists in fungi, and ultimately, whether the putative *H. capsulatum* OGA1 enzyme alters *O*-GlcNAcylation levels to influence morphogenesis. Interestingly, while homologs of genes that mediate *O*-GlcNAc signaling in metazoans (*OGT* and *OGA1*) can be found in *H. capsulatum*, they are conspicuously absent from *S. cerevisiae* and *C. albicans* [44], making *H. capsulatum* a useful model system in which to study *O*-GlcNAc signaling. Overall, it will be important to understand how GlcNAc controls cells fate determination in fungi, as this may contribute to our understanding of the role of GlcNAc in cell signaling and developmental processes across all kingdoms of life.

Our study and other recent work in fungi provide insights into the regulation of intracellular GlcNAc levels. In most cells, GlcNAc is thought to primarily exist as UDP-GlcNAc [45], [46], which is the universal nucleotide-sugar donor for glycan biosynthesis. Most eukaryotic cells have long been thought to salvage GlcNAc from glycans in lysosomal compartments and synthesize UDP-GlcNAc *de novo* via the hexosamine pathway [45], as opposed to salvaging free, extracellular GlcNAc across their plasma membranes using dedicated carbohydrate transporters [47]. However, the recent discovery of functional plasma membrane GlcNAc transporters in fungi suggests that at least some eukaryotic cells can take up extracellular GlcNAc via a transporter-mediated process. Interestingly, homologs of Ngt1 can be found in some metazoans, including humans [24], and it remains to be investigated whether these homologs represent functional GlcNAc transporters. The implications of extracellular, transporter-mediated uptake of GlcNAc in eukaryotic cells changes our understanding of the overall cellular flux and regulation of the essential metabolite UDP-GlcNAc.

Our work highlights that some fungi possess multiple MFS GlcNAc transporters, Ngt1 and Ngt2, potentially allowing these organisms to more precisely control levels of intracellular GlcNAc and thus, UDP-GlcNAc. MFS transporters can exist either as oligomers or monomers in the plasma membrane; however, the oligomeric state for most characterized MFS transporters is unknown [48]. We hypothesize that the *H. capsulatum* Ngt1 and Ngt2 GlcNAc transporters could be acting (1) in cooperation as a heterooligomeric complex to transport GlcNAc; (2) with complementary functions, *e.g.*, one directly senses GlcNAc and the other transports GlcNAc; or (3) as transporters with different affinities for GlcNAc. Consistent with this latter hypothesis, it was recently proposed that yeast utilize dual-transporter systems with differing affinities for the same substrate to optimize nutrient homeostasis when environmental resources fluctuate [49].

Interestingly, only a subset of fungi that have Ngt1 transporters also have Ngt2. Additionally, in many of the fungi with both transporters, Ngt1 is located in the same genomic regions as other GlcNAc catabolic genes, whereas that is not the case for fungal species that only have Ngt1. Perhaps this differential genomic location reflects the need for alternate transcriptional regulation of GlcNAc utilization genes in organisms that have both Ngt1 and Ngt2. In support of this idea, our data indicates that GlcNAc utilization genes (*NGT1*, *HXK1*, *NAG1*, and *DAC1*) in *H. capsulatum* are not repressed by glucose, which is in contrast to *C. albicans* GlcNAc utilization genes that are highly repressed by glucose. Furthermore, many of the fungi with identifiable Ngt2 transporters are known human fungal pathogens (*H. capsulatum*, *B. dermatitidis*, *Coccidioides* spp., *T. verrucosum*, *A. fumigatus*, and *P. marneffei*), suggesting that Ngt2 might play a role in pathogenesis. Of note, GlcNAc can be utilized as a carbon source *in vivo* by some mammalian pathogens and commensals including the parasite *Leishmania major* that resides in macrophage phagolysosomes [50], the commensal bacteria *Escherichia coli* that resides in the gastrointestinal tract [51], and the bacterial pathogen *Salmonella enterica* serovar Typhimurium, which is found intracellularly within macrophage vacuoles [52]. It is hypothesized that these microbes, each of which occupies a very different niche within its host, are able to acquire host GlcNAc intracellularly from glycans being recycled within cellular lysosomal and endosomal compartments [45] or extracellulary from mucins [53]. It will be interesting to determine whether Ngt1 and Ngt2 play a role in nutrient acquisition during *H. capsulatum* macrophage colonization and growth.

One of the most intriguing observations from this work is that Ngt1 and Ngt2 are necessary for efficient morphogenesis in the absence of exogenous GlcNAc. Thus, we hypothesize that Ngt1 and Ngt2 may be sensing levels of extracellular, endogenous GlcNAc to monitor population density and/or signal filamentous growth. It has long been appreciated that microbial cells can regulate their growth in response to changing local environmental conditions as well as fluctuations within their own population density via autoregulatory factors [54]. In fungi, a handful of autoregulatory small molecules and peptides that control density-dependent growth or morphology, interspecies communication, and biofilm formation have been proposed [55]. As the building block of the fungal cell wall polysaccharide chitin, GlcNAc fits the definition of a small molecule that could serve as an autoregulatory factor as its extracellular concentration would be proportional to the number of actively dividing cells due to the extensive remodeling of chitin that accompanies fungal cell division. Since chitin appears to be a more broadly distributed component of the cell wall in filamentous fungi [56] as compared to yeast cells, which primarily accumulate chitin around septa and bud scars [57], it is compelling to speculate that GlcNAc levels could regulate multicelluar filamentous growth. Furthermore, GlcNAc may trigger other changes beyond morphogenesis as it has been implicated as a signal in interspecies communication in the Gram-negative bacterial pathogen *Pseudomonas aeruginosa*. *P. aeruginosa* utilizes a two-component response regulator to sense environmental GlcNAc (one source proposed is GlcNAc shed from peptidoglycan of Gram-positive bacteria) to control the production of an antimicrobial factor [58], [59].

Notably, a major conclusion of our work is that widely diverged fungal species are capable of responding to GlcNAc to initiate filamentous growth. GlcNAc-induced filamentation has been observed previously [60] in some members of the Saccharomycetes fungal class (including *C.albicans*, *Candida lusitaniae*, and *Yarrowia lipolytica*), which are much more closely related to each other than to thermally dimorphic fungi (including *H. capsulatum* and *B. dermatitidis* from the Eurotiomycetes class) [61]. Equally noteworthy is that *C. albicans* and thermally dimorphic fungi occupy disparate environmental niches: *C. albicans* colonizes the mammalian gut and forms filaments at mammalian body temperature during invasive, pathogenic growth whereas thermally dimorphic fungi form filaments in the soil at the ambient environmental temperature. Thus, in spite of the distinct biological milieu occupied by these organisms, filamentation of fungal cells in response to exogenous GlcNAc appears to be deeply conserved, and therefore is likely to play a fundamental role in fungal biology.

Finally, we note that the life cycle of thermally dimorphic fungal pathogens, which includes the ability to switch between a parasitic form (disease-causing state) and a multicellular filamentous form (infectious state), is crucial to their pathogenesis and infectivity. Temperature is the best characterized cue that governs this reversible morphogenesis; however, as we demonstrated with GlcNAc, additional signals facilitate the efficient morphogenesis of thermally dimorphic fungi. It is critical to define the regulatory networks that integrate multiple environmental cues (*i.e.*, GlcNAc and temperature) into a morphogenetic program for the cellular differentiation of these organisms. Ultimately, understanding how *H. capsulatum* yeast cells transition to filaments will provide insight into the establishment and maintenance of the infectious environmental reservoir of this human fungal pathogen.

## Materials and Methods

### Strains and culture conditions

*Histoplasma capsulatum* strains G217B (ATCC26032), G217B*ura5*Δ (WU15), both gifts from the laboratory of William Goldman, University of North Carolina, Chapel Hill, were grown in HMM (Histoplasma-macrophage medium) broth or plates [62]. *Blastomyces dermatitidis* strain SLH14081 (gift of Bruce Klein, University of Wisconsin, Madison) was grown in HMM broth. HMM medium, which contains 110 mM glucose, was supplemented when necessary with uracil (Sigma-Aldrich) (200 µg/ml) or as indicated with GlcNAc (Sigma-Aldrich) (referred to as HMM medium supplemented with the mM concentration of GlcNAc indicated throughout the text). In some experiments, GlcNAc was used as the major carbohydrate source in HMM broth and plates by substituting 100 mM GlcNAc in place of the usual major carbon source, 100 mM glucose, to provide an equal molarity of carbon source (referred to as “HMM/100 mM GlcNAc” throughout the text); however all HMM medium retains 10 mM glucose from the Gibco's F12 nutrient supplement (Life Technologies) that is used to make HMM medium [62]. *H. capsulatum* and *B. dermititidis* cultures were grown at 37°C under 5% CO_2_ for yeast-phase growth or at room temperature (RT) for filamentous-phase growth with continuous shaking of liquid cultures on an orbital shaker.

For the microarray time-course study, G217B yeast cells were grown at 37°C in HMM medium and subjected to passage at 1∶25 dilution into HMM medium. After 1 day of growth to early log phase, a portion of cells were harvested for the t = 0 timepoints and the remaining cells were washed in PBS and then resuspended with no dilution into 200 mL HMM (contains 110 mM glucose) and HMM/100 mM GlcNAc (contains 10 mM glucose and 100 mM GlcNAc) media for t = 1 h, 4 h, and 24 h timepoints or resuspended with a 1∶10 dilution into 200 mL HMM (contains 110 mM glucose) and HMM/100 mM GlcNAc media (contains 10 mM glucose and 100 mM GlcNAc) for t = 4 d and 7 d timepoints. Cultures for all timepoints were allowed to continue to grow at 37°C under 5% CO_2_ for yeast-phase growth or transferred to RT for filamentous-phase growth. For the endpoint microarray experiment, G217B yeast cells were grown for 2 days at 37°C in HMM medium and filamentous cells were grown for 4–6 weeks with passaging 3 times (1∶5 dilution) into fresh HMM medium at RT before reaching a sufficient density of cells for harvesting. At each indicated timepoint, cultures were harvested and processed as described below.

For quantitative reverse transcriptase PCR (qRT-PCR), strains were grown at 37°C in 5 mL HMM medium to log phase. Cultures were synchronized to reach early-log phase (OD_600_ = 4.0–6.0) the day of the experiment. Cells were washed once in PBS, and a 1∶10 dilution of each strain was inoculated into HMM (110 mM glucose) and HMM/100 mM GlcNAc (contains 10 mM glucose and 100 mM GlcNAc) media. After 2 days of growth, cultures were harvested by centrifugation and total RNA was harvested using a guanidine thiocyanate lysis protocol as previously described [11].

### Construction of RNAi strains

A 457 bp region of *NGT1*, 517 bp region of *NGT2*, and a 464 bp region of the *HXK1* coding sequences were amplified using G217B cDNA and oligonucleotides OAS2880-81, OAS2876-77, or OAS4193-94, respectively. All primer sequences are included in Table S7. Using Gateway cloning and the entry vector pDONR/zeo (Life Technologies), these PCR products were used to generate BAS662 containing a hairpin repeat of *NGT1* and BAS1198 containing a hairpin repeat of *HXK1* in backbone vector pFANTAi4 (gift of Bruce Klein, University of Wisconsin, Madison; [63]) or BAS643 containing a hairpin repeat of *NGT2* in vector pSB23 (a Gateway-compatible destination plasmid derived from pCR186, which was a gift from Chad Rappleye, Ohio State University). The vector control (BAS506), BAS662 (*NGT1* RNAi), and BAS1198 (*HXK1* RNAi) constructs were integrated into *H. capsulatum* strain G217B*ura5*Δ by the use of an Agrobacterium-mediated gene transfer method as described previously [12], [64]. The episomally-maintained vector control (BAS538) and BAS643 (*NGT2* RNAi) were electroporated into G217B*ura5*Δ as previously described [31]. The method chosen for RNAi plasmid maintenance (*i.e.*, episomal versus integrating) was determined empirically by assessing which method gave the strongest and most consistent decrease of *NGT1* and *NGT2* expression.

### Confocal imaging time-courses

Yeast form cultures of SLH14081, G217B*ura5*Δ, vector control, *NGT1*, or *NGT2* RNAi strains were grown to early log phase at 37°C in HMM medium, washed once in PBS and sonicated for 3 s to disperse clumps. For the *H. capsulatum* G217B*ura5*Δ and *B. dermititidis* SLH14081 wild-type time-course, 10 µl of 5×10^6^ yeast cells/mL (G217B*ura5*Δ) or 10 µl of 1×10^6^ yeast cells/mL (SLH14081) were loaded into 28 mm×120 µm M04S CellASIC microfluidic cell culture plates (Millipore) in HMM (contains 110 mM glucose) or HMM/100 mM GlcNAc (contains 10 mM glucose and 100 mM GlcNAc) medium and transferred to RT to monitor conversion to filamentous cells or to 37°C to monitor yeast form growth. For the *NGT* and *HXK1* RNAi time courses, 10 µl of 5×10^6^ yeast cells/mL of *H. capsulatum NGT1* RNAi, *NGT2* RNAi, *HXK1* RNAi, or vector control strains were loaded into 28 mm×120 µm M04S CellASIC microfluidic cell culture plates (Millipore) in HMM medium (contains 110 mM glucose) and HMM medium supplemented with 10 mM GlcNAc (contains 110 mM glucose and 10 mM GlcNAc). At each indicated timepoint, cell morphology was examined using live-cell differential interference contrast (DIC) microscopy with 65, 1.2 µm-thick Z-stack images acquired using a Yokogawa CSU-X1 spinning disk confocal mounted on a Nikon Eclipse Ti inverted microscope with an Andora Clara digital camera and a CFI APO TIRF 60× oil or PLAN APO 40× objective. Images were acquired by and processed in NIS-Elements software 4.10 (Nikon). For the *NGT* RNAi yeast to filament transition experiment, the number of yeast cells remaining at each timepoint was scored by counting the total number of yeast and filamentous cells visible in a maximum intensity projection of the Z-stack image covering a 37.5×37.5 µm area. Images with no visible yeast cells (i.e., only filamentous cells present) were scored as 0% yeast cells.

### Assessment of G217B yeast and filamentous growth in various carbon sources

Yeast form cultures of G217B were grown to early log phase in HMM medium at 37°C. 10-fold serial dilutions of G217B yeast cells were spotted onto HMM solid medium containing 110 mM glucose in the absence (110 mM glucose only) or presence of 0.1 mM, 0.25 mM, or 1 mM GlcNAc and HMM/100 mM GlcNAc (contains 10 mM glucose and 100 mM GlcNAc) and transferred to RT to monitor filamentous growth or grown at 37°C to monitor yeast phase growth. To monitor growth on various carbon sources, cells were spotted onto HMM solid medium containing 110 mM glucose in the absence or presence of 1 mM GlcNAc, 1 mM fructose (Sigma-Aldrich), or 1 mM glucosamine (Sigma-Aldrich). Cells were also analyzed in liquid medium by inoculating a 1∶10 dilution of G217B yeast cells into 10 mL of HMM medium containing 110 mM glucose and supplemented with 10 mM GlcNAc or 10 mM glucose (Sigma-Aldrich) and then incubated at RT to monitor the transition to filamentous cells or at 37°C to monitor yeast phase growth. Dilution series on plates as well as liquid cultures were monitored for growth and morphology by light microscopy between 6 and 14 days after inoculation.

### BLASTP and phylogenetic analysis

*H. capsulatum* homologs to the *C. albicans* GlcNAc catabolic genes (*NGT1* = orf19.5392; *HKX1* = orf19.2154; *DAC1* = orf19.2157; and *NAG1* = orf19.2156; gene identities from http://www.candidagenome.org/) were identified by BLASTP [65]. For phylogenetic analysis, homologs of *H. capsulatum* Ngt1 and Ngt2 in each indicated fungal species were identified by BLASTP using a cut-off value of E≤1×10^−5^. After alignment of protein sequences with MUSCLE [66], an unrooted phylogenetic model was generated using MrBayes [67]. NCBI protein accession numbers are given in Table S5 and Figure S8.

### Microarrays

Cells were harvested by centrifugation or filtration and total RNA was isolated using a guanidine thiocyanate lysis protocol as previously described [11]. Fluorescently labeled cDNA was synthesized by incorporating amino-allyl dUTP during reverse transcription with Superscript II (Life Technologies) of 15 µg total RNA with oligonucleotide-dT and random hexamers used as primers. Cy3 or Cy5 dyes (GE Life Sciences) were coupled to the amino-allyl group as described previously [68]. For each time-course sample, cDNA was coupled to Cy5 and a reference cDNA pool was made by combining RNA from t = 0 and all late time course samples, which was coupled to Cy3. For end point microarray experiments (*i.e.*, established yeast samples compared to established filamentous samples), G217B yeast cDNA was coupled to Cy5 and filament cDNA was coupled to Cy3. Samples were hybridized to *H. capsulatum* G217B 70-mer oligonucleotide microarrays. Each microarray contained one or two 70-mer oligonucleotides for each predicted gene in the G217B genome (11,088 gene predictions and a total of 14,820 oligonucleotides per array). Arrays were scanned on a GenePix 4000B scanner (Axon Instruments/Molecular Devices) and analyzed using GenePix Pro, version 6.0 (Molecular Devices), NOMAD 2.0 (http://derisilab.ucsf.edu/microarray/software.html), Cluster 3.0 [69], and Java Treeview 1.1.4r4 (available at http://jtreeview.sourceforge.net). To eliminate elements with low signal, we analyzed only elements for which the sum of the medians for the 635 nm and 532 nm channels was ≥150 intensity units. Gene expression data was filtered for 80% completion and a cut-off value for change in gene expression of >2.0 (log_2_) for all clusters is shown unless otherwise indicated. Hierarchical or k-means clustering were used as indicated for unsupervised clustering. For k-means clustering, k = 10 and n = 100 parameters were empirically chosen. All microarray data have been deposited at Gene Expression Omnibus (GEO) database at the National Center for Biotechnology Information (http://www.ncbi.nlm.nih.gov/geo/) and are available through the accession number GSE48044.

### Growth assessment of *NGT1*, *NGT2*, and *HXK1* RNAi strains

Yeast form cultures of *NGT1* RNAi, *NGT2* RNAi, *HXK1* RNAi and corresponding vector control strains were grown in HMM medium at 37°C and synchronized to mid log phase for the day of the experiment. Cells were washed in PBS and then resuspended in 3M minimal medium [62] containing no carbohydrate (without glucose) and grown overnight to starve cells. After starvation, cells were inoculated to an OD_600_ = 0.6 into 3M medium containing equal molarities of glucose or GlcNAc (55 mM), or no carbohydrate. Growth was monitored by measuring the OD_600_ at each indicated timepoint.

### Quantitative RT-PCR

Total RNA was treated with DNase I (Promega). cDNA was synthesized using 3.3 µg of DNase I-treated RNA, Affinityscript reverse transcriptase (Agilent), and oligo(dT) and random hexamer primers. qRT-PCR was performed using 1∶100 dilutions of cDNA, 0.8× FastStart Universal SYBR Green Master Mix (Roche), and 200 nM primers. Reactions were performed using an Mx3000P qPCR system (Agilent) with the Comparative Quantitation program. Cycling parameters were 95°C for 10 min and then 40 cycles of 95°C (30 s) and 55°C (1 min); cycling was followed by dissociation curve analysis. Reactions were analyzed using MxPro software (Agilent). Primer sequences of each qRT-PCR probe are included in Table S7.

### Heterologous expression of *H. capsulatum NGT1* and *NGT2* in *C. albicans*

Coding sequences of *H. capsulatum NGT1* and *NGT2* were codon optimized by gene synthesis (GENEWIZ) for expression in *C. albicans* using the most frequent *S. cerevisiae* codon and excluding use of the CUG codon (to avoid translation of CUG as serine instead of the canonical leucine which occurs *C. albicans* [70]). Plasmids for complementation were constructed using PCR and homologous recombination in *S. cerevisiae* [71]. *H. capsulatum* codon optimized *NGT1* and *NGT2* were put under the control of the *C. albicans TDH3* promoter and *ACT1* terminator followed by the *C. albicans URA3* gene. A vector control construct was made identically except it lacked *NGT1* or *NGT2* coding sequence. All primers are listed in Table S7. Complementation fragments containing ∼350 bp of homology to *RPS10* were excised by digestion with PmeI and then integrated into the *C. albicans ngt1*Δ strain at the *RPS10* locus via homologous recombination. Strains were verified by PCR to contain the appropriate *NGT* gene and then 10-fold dilutions of cells were spotted onto solid Yeast Nitrogen Base minimal medium containing 50 mM of GlcNAc, glucose, or galactose. Growth results were reproducible with separate isolates obtained from independent transformations. *C. albicans* strains used in this study are listed in Table S8.

Supplementary Materials and Methods can be found in Text S1.

## Supporting Information

Figure S1 GlcNAc-promoted morphogenesis is not due to simply providing an additional carbon source. *H. capsulatum* yeast cells were inoculated into liquid HMM medium (containing 110 mM glucose) supplemented with either 10 mM glucose or 10 mM GlcNAc and transferred to RT for filamentous growth. Cellular morphology was assessed by light microscopy after 6 days of growth at RT. Scale bar, 20 µm. Representative images are shown.
(TIF)

Figure S2 Growth rates of *H. capsulatum* cells at 37°C and RT. (A) *H. capsulatum* yeast cells grow slowly at RT before their conversion to filaments irrespective of the presence of GlcNAc. *H. capsulatum* yeast cells were inoculated into liquid HMM (glucose), HMM/100 mM GlcNAc, or HMM+10 mM GlcNAc medium and transferred to RT. At each indicated timepoint after transition to RT, cells were imaged by DIC confocal microscopy. Yeast cells and yeast cells beginning to transition into filaments were quantified by counting to score the number of budding cells. For the GlcNAc 72 h timepoints, no data are shown since cells have converted to filaments by this timepoint. The standard deviation of the mean number of cells at each timepoint from three biological replicates is shown. (B) Schematic of morphological differentiation and growth of *H. capsulatum* yeast cells after transition to RT as quantified in panel A. Yeast cells grow slowly at RT before conversion to filaments. Glucose-grown cells experience slightly more yeast cell division at RT over time, which seems to correlate with slower conversion to filaments. (C) GlcNAc promotes faster growth of *H. capsulatum* filaments. *H. capsulatum* filaments generated in glucose medium at RT were plated onto HMM (glucose), HMM/100 mM GlcNAc, or HMM+10 mM GlcNAc medium and allowed to continue to grow at RT. At each indicated timepoint, the colony diameter was measured to assess the rate of filamentous cell growth at RT. The standard deviation of the mean colony diameter at each timepoint from four biological replicates is shown.
(EPS)

Figure S3 Morphology of *H. capsulatum* during yeast-to-filament transcriptional profiling. (A) Morphology of *H. capsulatum* yeast cells transitioning to filaments at RT in HMM glucose or HMM/100 mM GlcNAc medium as assessed by light microscopy for the duration of the transcriptional profiling time course. (B) Cellular morphology of *H. capsulatum* yeast cells grown in either HMM glucose or HMM/100 mM GlcNAc medium at 37°C from day 7 microarray timepoint as assessed by light microscopy. Representative images are shown.
(TIF)

Figure S4 P*_TYR1_*-GFP is induced earlier in GlcNAc-grown yeast cells transitioning to filaments. *H. capsulatum* yeast cells carrying a GFP reporter construct under the control of approximately 1 kb of the *TYR1* promoter were switched from 37°C to RT for filamentous growth in the presence (A, HMM/100 mM GlcNAc) or absence (B, HMM glucose) of exogenous GlcNAc. GFP fluorescence (shown in green, 488 nm laser) and DIC images were taken at the indicated timepoints. Maximum intensity projections are depicted for each Z-stack image. Scale bar, 10 µm. Representative images are shown.
(TIF)

Figure S5 GlcNAc utilization in fungi. Pathway of GlcNAc transport and utilization in fungi. Extracellular GlcNAc is taken up via a GlcNAc-specific plasma membrane transporter, Ngt, where it becomes phosphorylated by the kinase Hxk1 to form GlcNAc-6P. GlcNAc-6P can then either be catabolized as an energy source or converted into the universal nucleotide GlcNAc donor, uridine diphosphate (UDP)-GlcNAc (enzymatic steps not shown), for use in glycan biosynthesis. When being catabolized by cells, GlcNAc-6P becomes deacetylated by the GlcNAc-6P deacetylase Dac1 to form Glucosamine-6P, which is further deaminated and isomerized by Nag1 into Fructose-6P for use in glycolysis.
(EPS)

Figure S6 Schematic of the genomic organization of GlcNAc utilization genes. Examples of the genomic organization of GlcNAc utilization genes in a subset of fungal species. As shown, *NGT1* clusters near GlcNAc utilization genes in the genomes of *H. capsulatum*, *B. dermititidis*, and *Aspergillus fumigatus*. This is in contrast to *C. albicans NGT1* (located on chromosome 3), which is not located near the *C. albicans* GlcNAc utilization gene cluster in the genome (located on chromosome 6). For simplicity, only predicted GlcNAc utilization genes in each genomic region are shown and their contig or chromosomal location is indicated for each species. See Table S6 for gene identities and genomic coordinates.
(EPS)

Figure S7 Phylogenetic tree demonstrating the evolutionary conservation of *H. capsulatum* Ngt1 and Ngt2 homologs. BLASTP was used to identify homologs to *H. capsulatum* Ngt1 and Ngt2. All BLASTP matches (E≤1×10^−5^) from the genomes of 20 sequenced Ascomycetes and Basidiomycetes species were aligned using MUSCLE. A phylogenetic tree was built with aligned sequences using MrBayes. Protein IDs are indicated next to each species name as NCBI accession codes. The Ngt1/Ngt2 clade is highlighted.
(EPS)

Figure S8 RNAi depletion of *H. capsulatum NGT1*, *NGT2*, and *HXK1* transcripts. *H. capsulatum* vector control, *NGT1* RNAi, *NGT2* RNAi, and *HXK1* RNAi strains were grown in glucose medium and levels of *NGT1* (A) *NGT2* (B) and *HXK1* (C) transcripts were assessed for each strain by qRT-PCR. Percentage of *NGT1*, *NGT2*, or *HXK1* expression is shown normalized to *ACT1* and is relative to vector control expression levels (set to 100%). The standard deviation of mean expression values from three independent RNAi clones for each knockdown construct is shown.
(EPS)

Figure S9 *NGT1* influences the expression of GlcNAc-inducible transcripts during normal cellular growth. *H. capsulatum NGT1* vector control, *NGT2* vector control, *NGT1* RNAi, and *NGT2* RNAi strains were grown in glucose medium and levels of *NAG1* (A), *DAC1* (B), *OGA1* (C), and *PTR2* (D) transcripts were assessed for each strain by qRT-PCR. Expression is shown normalized to *ACT1* and the standard deviation of mean expression values from three independent RNAi or vector control clones for each knockdown construct is shown.
(EPS)

Figure S10 *NGT1* and *NGT2* influence the expression of GlcNAc-induced transcripts not predicted to be involved in GlcNAc catabolism. *NGT1* and *NGT2* transcript levels were depleted by RNAi in *H. capsulatum* and qRT-PCR was used to determine levels of *OGA1* and *PTR2* transcripts in each knockdown strain as compared to vector control cells in the absence or presence of GlcNAc (100 mM). Transcript levels were normalized to *ACT1*. The standard deviation of mean expression values from three independent RNAi and vector control clones is shown.
(EPS)

Figure S11 *H. capsulatum NGT1* and *NGT2* restore GlcNAc-promoted filamentation of the *C. albicans NGT1* mutant. Expression of *H. capsulatum NGT1* or *NGT2* restores GlcNAc-induced filamentation of *C. albicans ngt1*Δ. *H. capsulatum NGT1* (Hc*NGT1*), *H. capsulatum NGT2* (Hc*NGT2*), *C. albicans NGT1* (Ca*NGT1*), or empty vector (EV) were introduced into *C. albicans ngt1*Δ yeast cells (*ngt1*Δ). The morphology of transformants was examined by light microscopy after 3 hours of growth at 37°C in liquid medium containing 2.5 mM glucose or 2.5 mM GlcNAc and compared to the morphology of wild-type *C. albicans* (WT). Representative images are shown.
(TIF)

Figure S12 GlcNAc catabolism and utilization are not required for *H. capsulatum* GlcNAc-promoted filamentation. (A) *HXK1* is necessary for growth of *H. capsulatum* in medium where GlcNAc is the major carbon source. Vector control and *HXK1* RNAi strains were starved overnight to deplete available carbon sources and then inoculated into 3M minimal medium containing glucose, GlcNAc, or no carbohydrate. At each indicated timepoint, growth was evaluated by measuring the optical density at 600 nm. The standard deviation of mean OD_600_ values from three independent *HXK1* RNAi and vector control clones is shown. (B,C) *HXK1* RNAi strains filament in response to GlcNAc. Vector control and *HXK1* RNAi yeast cells grown at 37°C were inoculated into HMM medium supplemented with 10 mM GlcNAc (B) or HMM medium (C, glucose) and transferred to RT to monitor conversion to filaments. Cell morphology was assessed at each indicated timepoint by confocal DIC microscopy on live cells. Scale bar, 10 µm. Representative images are shown.
(TIF)

Figure S13 Chitin promotes morphogenesis of *H. capsulatum* at RT. *H. capsulatum* yeast cells grown at 37°C were inoculated into liquid HMM medium (glucose), HMM medium containing 100 ng/mL of GlcNAc, or HMM medium containing 100 ng/mL of chitin and transferred to RT to monitor conversion to filaments. Cell morphology was assessed at each indicated timepoint by confocal DIC microscopy on live cells. Scale bar, 10 µm. Representative images are shown.
(TIF)

Table S1 Tab delimited text file of the k-means cluster demonstrating temporally regulated transcripts during morphogenesis shown in Figure 3B.
(TXT)

Table S2 Gene identities of transcripts shown in Figure 4. *H. capsulatum* G217B strain formal gene names, abbreviated gene names, and annotations are given for all genes shown in Figure 4. Gene and sequence information are available at http://histo.ucsf.edu/.
(XLS)

Table S3 Tab delimited text file of hierarchical clustering.
(TXT)

Table S4 Gene identities of transcripts shown in Figure 5. *H. capsulatum* G217B strain formal gene names, abbreviated gene names, and annotations are given for all genes shown in Figure 5. Gene and sequence information are available at http://histo.ucsf.edu/.
(XLS)

Table S5 Protein accession numbers of Ngt1 and Ngt2 from Figure 6. NCBI protein accession numbers for Ngt1 and Ngt2 homologs shown in Figure 6 or G217B formal gene name (G217B sequence information available at http://histo.ucsf.edu/).
(XLS)

Table S6 Gene identities of GlcNAc catabolic genes shown in Figure S6. Formal genes names, abbreviated gene names, and chromosomal coordinates are given for GlcNAc catabolic genes shown in Figure S7. (G217B sequence information available at http://histo.ucsf.edu/).
(XLS)

Table S7 Primer sequences used in this study. Hc = *H. capsulatum*, Ca = *C. albicans*.
(XLS)

Table S8 *C. albicans* strains used in this study. Hc = *H. capsulatum*, Ca = *C. albicans*.
(XLS)

Text S1 Supplementary materials and methods.
(DOC)
