# Supplementary figures and images for: N-acetylglucosamine (GlcNAc) Triggers a Rapid, Temperature-Responsive Morphogenetic Program in Thermally Dimorphic Fungi
Source: PLoS Genet. 2013 Sep 19;9(9):e1003799. doi: 10.1371/journal.pgen.1003799 (PMC3778022; doi:10.1371/journal.pgen.1003799)

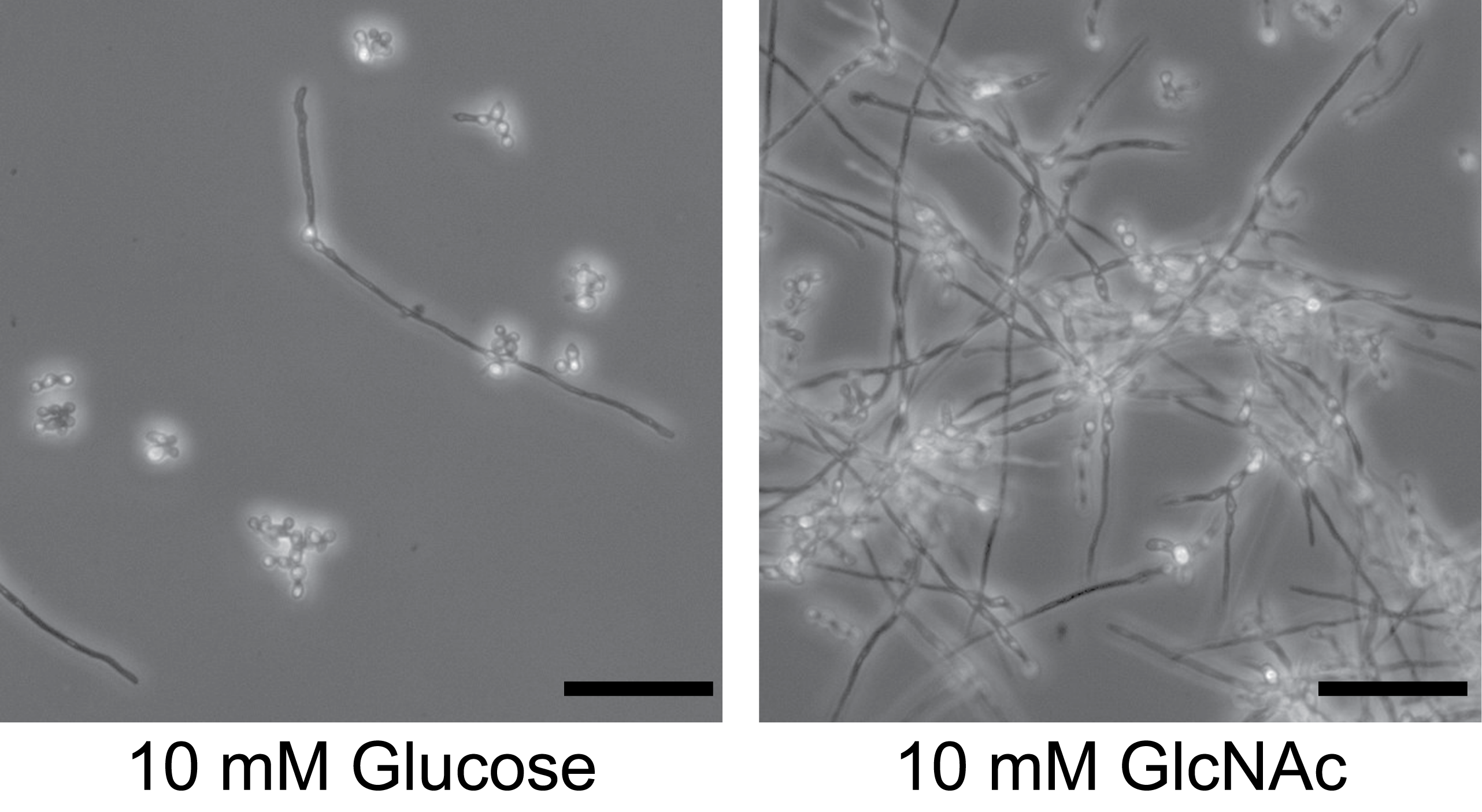

Supplement: Figure S1 — GlcNAc-promoted morphogenesis is not due to simply providing an additional carbon source. H. capsulatum yeast cells were inoculated into liquid HMM medium (containing 110 mM glucose) supplemented with either 10 mM glucose or 10 mM GlcNAc and transferred to RT for filamentous growth. Cellular morphology was assessed by light microscopy after 6 days of growth at RT. Scale bar, 20 µm. Representative images are shown. (TIF) [file pgen.1003799.s001.tif]

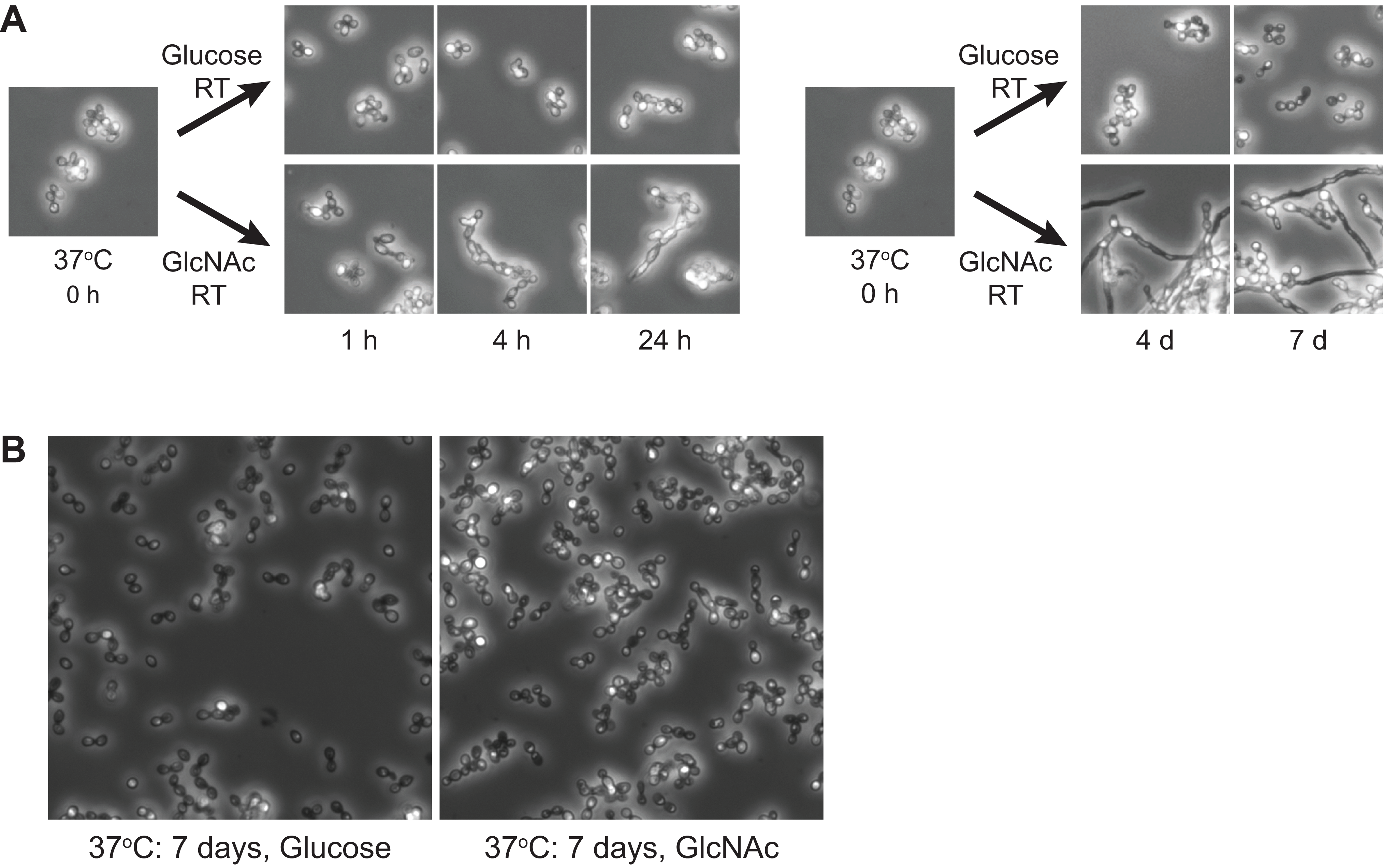

Supplement: Figure S3 — Morphology of H. capsulatum during yeast-to-filament transcriptional profiling. (A) Morphology of H. capsulatum yeast cells transitioning to filaments at RT in HMM glucose or HMM/100 mM GlcNAc medium as assessed by light microscopy for the duration of the transcriptional profiling time course. (B) Cellular morphology of H. capsulatum yeast cells grown in either HMM glucose or HMM/100 mM GlcNAc medium at 37°C from day 7 microarray timepoint as assessed by light microscopy. Representative images are shown. (TIF) [file pgen.1003799.s003.tif]

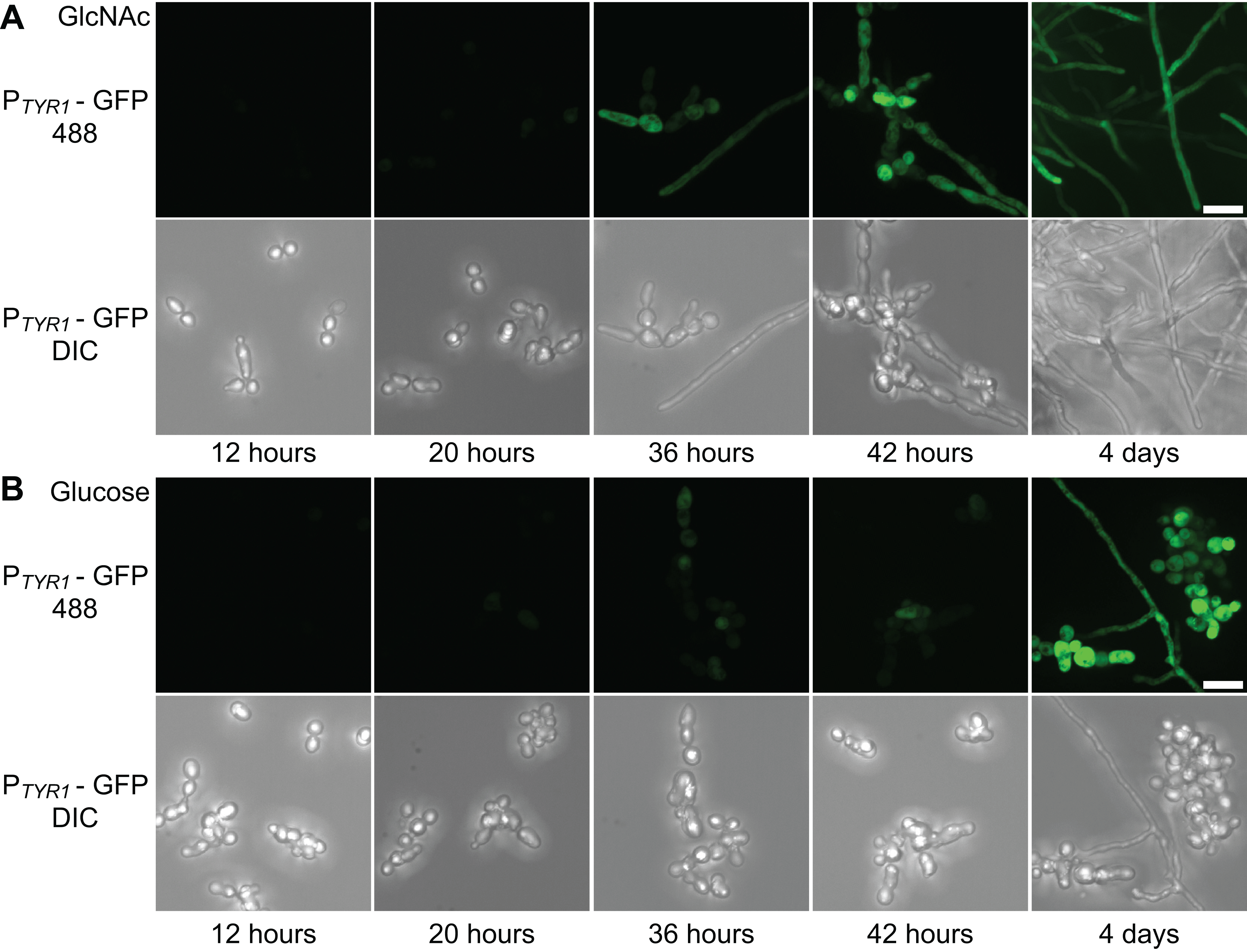

Supplement: Figure S4 — PTYR1-GFP is induced earlier in GlcNAc-grown yeast cells transitioning to filaments. H. capsulatum yeast cells carrying a GFP reporter construct under the control of approximately 1 kb of the TYR1 promoter were switched from 37°C to RT for filamentous growth in the presence (A, HMM/100 mM GlcNAc) or absence (B, HMM glucose) of exogenous GlcNAc. GFP fluorescence (shown in green, 488 nm laser) and DIC images were taken at the indicated timepoints. Maximum intensity projections are depicted for each Z-stack image. Scale bar, 10 µm. Representative images are shown. (TIF) [file pgen.1003799.s004.tif]

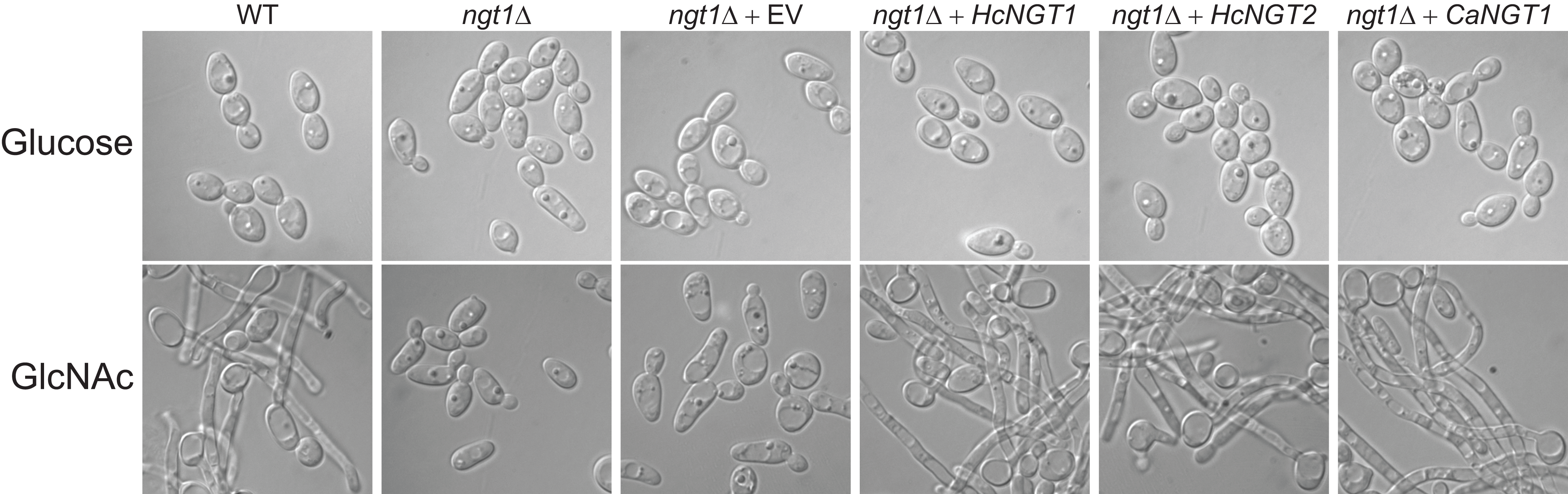

Supplement: Figure S11 — H. capsulatum NGT1 and NGT2 restore GlcNAc-promoted filamentation of the C. albicans NGT1 mutant. Expression of H. capsulatum NGT1 or NGT2 restores GlcNAc-induced filamentation of C. albicans ngt1Δ. H. capsulatum NGT1 (HcNGT1), H. capsulatum NGT2 (HcNGT2), C. albicans NGT1 (CaNGT1), or empty vector (EV) were introduced into C. albicans ngt1Δ yeast cells (ngt1Δ). The morphology of transformants was examined by light microscopy after 3 hours of growth at 37°C in liquid medium containing 2.5 mM glucose or 2.5 mM GlcNAc and compared to the morphology of wild-type C. albicans (WT). Representative images are shown. (TIF) [file pgen.1003799.s011.tif]

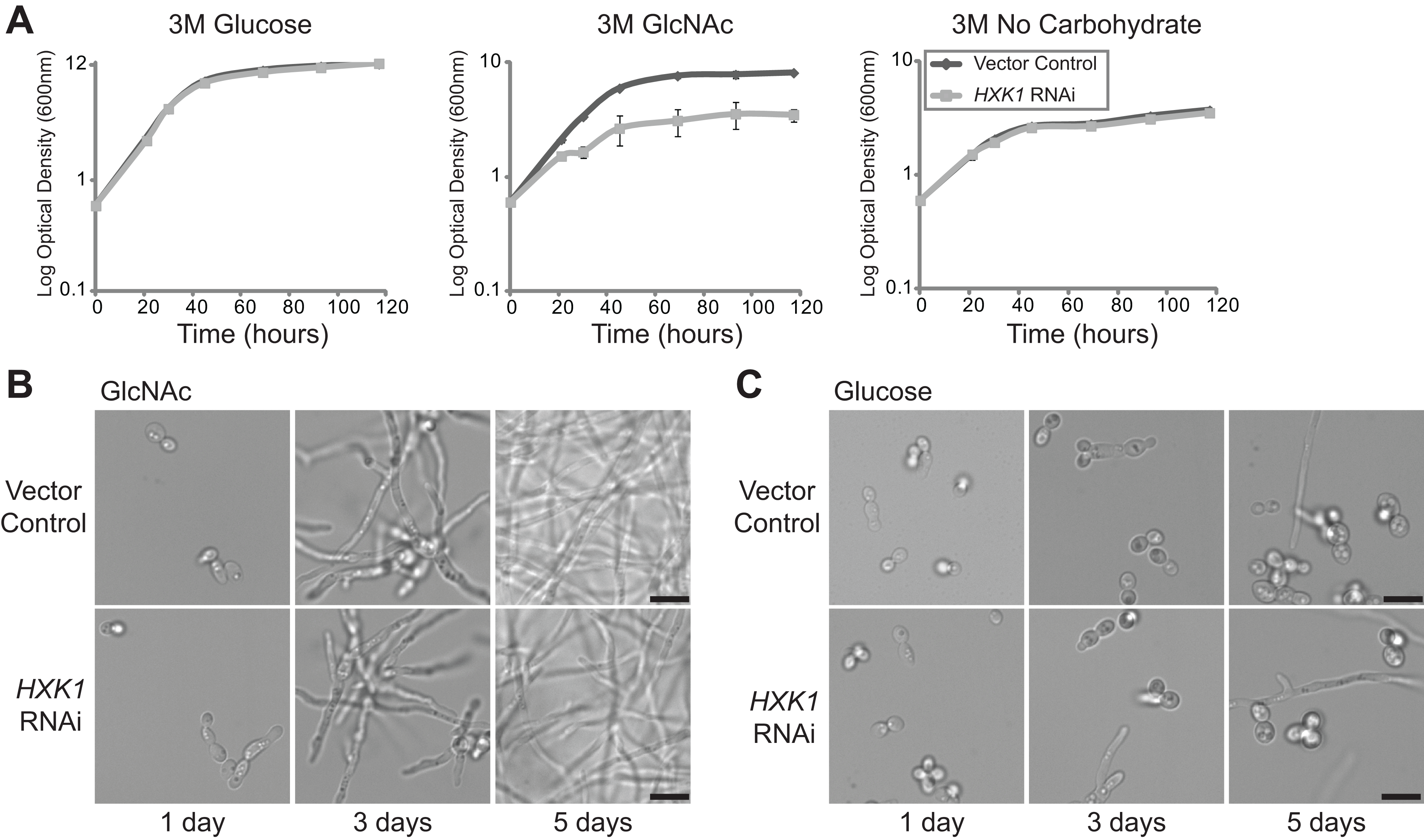

Supplement: Figure S12 — GlcNAc catabolism and utilization are not required for H. capsulatum GlcNAc-promoted filamentation. (A) HXK1 is necessary for growth of H. capsulatum in medium where GlcNAc is the major carbon source. Vector control and HXK1 RNAi strains were starved overnight to deplete available carbon sources and then inoculated into 3M minimal medium containing glucose, GlcNAc, or no carbohydrate. At each indicated timepoint, growth was evaluated by measuring the optical density at 600 nm. The standard deviation of mean OD600 values from three independent HXK1 RNAi and vector control clones is shown. (B,C) HXK1 RNAi strains filament in response to GlcNAc. Vector control and HXK1 RNAi yeast cells grown at 37°C were inoculated into HMM medium supplemented with 10 mM GlcNAc (B) or HMM medium (C, glucose) and transferred to RT to monitor conversion to filaments. Cell morphology was assessed at each indicated timepoint by confocal DIC microscopy on live cells. Scale bar, 10 µm. Representative images are shown. (TIF) [file pgen.1003799.s012.tif]

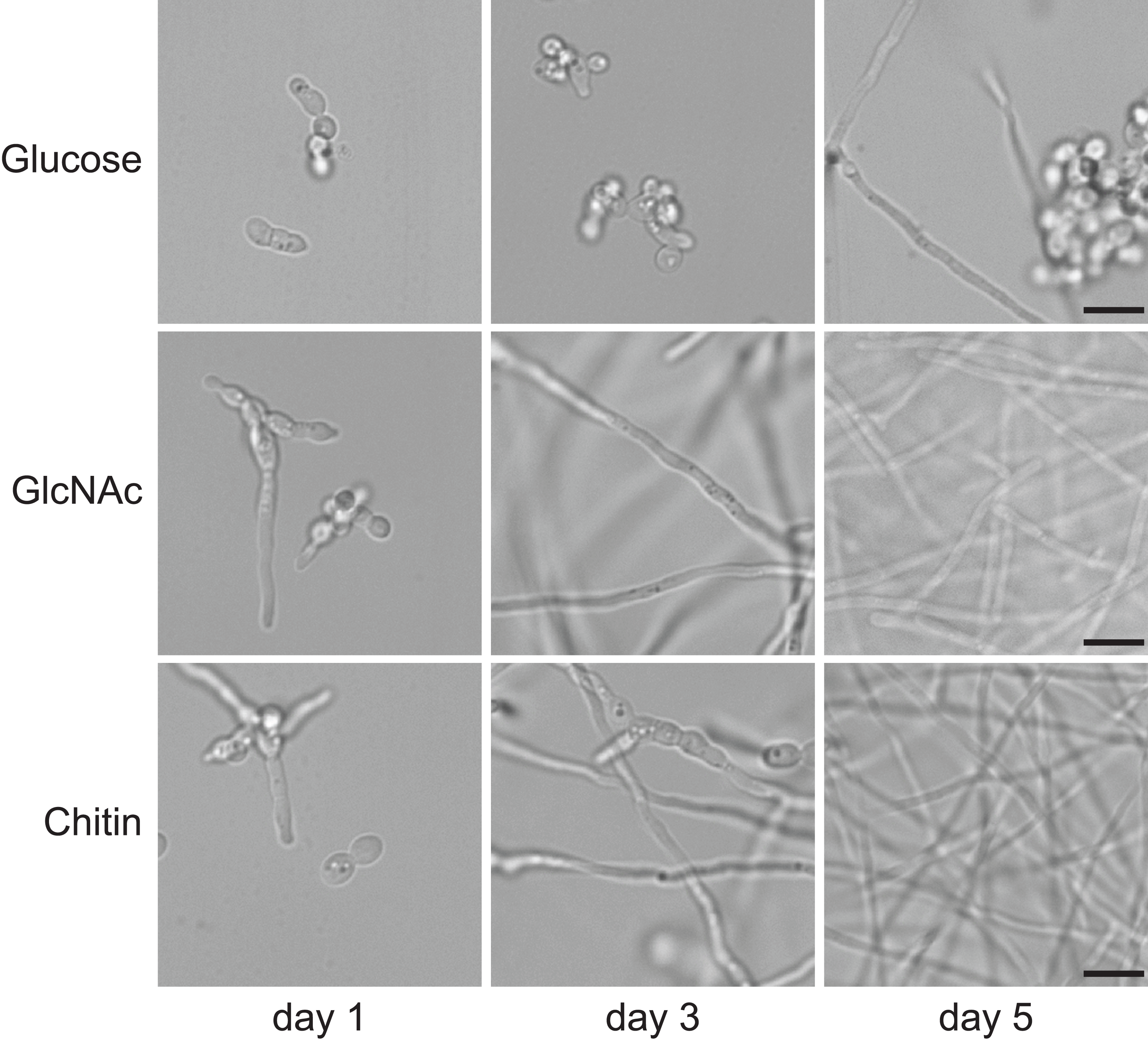

Supplement: Figure S13 — Chitin promotes morphogenesis of H. capsulatum at RT. H. capsulatum yeast cells grown at 37°C were inoculated into liquid HMM medium (glucose), HMM medium containing 100 ng/mL of GlcNAc, or HMM medium containing 100 ng/mL of chitin and transferred to RT to monitor conversion to filaments. Cell morphology was assessed at each indicated timepoint by confocal DIC microscopy on live cells. Scale bar, 10 µm. Representative images are shown. (TIF) [file pgen.1003799.s013.tif]
