# Supplementary material for: N-acetylglucosamine (GlcNAc) Triggers a Rapid, Temperature-Responsive Morphogenetic Program in Thermally Dimorphic Fungi
Source: PLoS Genet. 2013 Sep 19;9(9):e1003799. doi: 10.1371/journal.pgen.1003799 (PMC3778022; doi:10.1371/journal.pgen.1003799)
Supplement: Text S1 — Supplementary materials and methods. (DOC) [file pgen.1003799.s022.doc]

**Supplementary Materials and Methods**

**Measuring yeast-phase and filamentous-phase growth rates at RT**

To measure growth rates of yeast cells at RT prior to conversion to filaments, yeast form cultures of G217B*ura5*Δ were grown to early log phase at 37°C in HMM medium, washed once in PBS, and sonicated for 3 s to disperse clumps. Cells were adjusted to 5 x 10^5^ cells/mL in HMM (containing 110 mM glucose), HMM/100 mM GlcNAc (containing 100 mM GlcNAc and 10 mM glucose), or HMM supplemented with 10 mM GlcNAc (containing 110 mM glucose and 10 mM GlcNAc) medium and 100 µl of each cell suspension was seeded in triplicate into glass bottom 96 well plates (# 1.5 High Performance Cover Glass, 0.17 nm; In Vitro Scientific). Plates were briefly centrifuged to settle cells and placed at RT for growth. Cells were imaged at each indicated timepoint using live-cell DIC confocal microscopy as described in the main text Materials and Methods. The number of yeast cells was quantified at each timepoint by sampling 4 different 37.5 x 37.5 µm regions for each biological replicate. At least 100 cells per timepoint per biological replicate were counted. A yeast cell starting to transition into a filament (*i.e*., 24 h, 48 h GlcNAc timepoints and 72 h glucose timepoint) was counted as one budding cell. To measure growth rates of filamentous cells at RT, G217B *H. capsulatum* filamentous cells were grown at RT on Difco Sabouraud dextrose (BD Biosciences) agar, and harvested into PBS to make a suspension of filaments. 20 ul of the filament suspension was spotted in quadruplicate onto HMM (containing 110 mM glucose), HMM/100 mM GlcNAc (containing 100 mM GlcNAc and 10 mM glucose), or HMM supplemented with 10 mM GlcNAc (containing 110 mM glucose and 10 mM GlcNAc) agar medium and put at RT for filamentous growth. At each indicated timepoint, the diameter of the colony was measured to monitor radial growth of filamentous cells over time.

***TYR1p*-GFP reporter construct expression kinetics**

The Gateway compatible (Life Technologies) entry vector, pDI-99, was created by cloning approximately 1 kb of the G217B *H. capsulatum* *TYR1* promoter upstream of GFP with a *H. capsulatum* G217B *CATB* terminator. pDI-99 was recombined with pDI-116 (a Gateway compatible version of pRH5b ) to create BAS880. BAS880 was integrated into *H. capsulatum* strain G217B by the use of an *Agrobacterium*-mediated gene transfer method as described in the main text and transformants were selected with hygromycin. A mid-log phase culture of G217B P*_TYR1_*-GFP yeast cells grown at 37°C in HMM medium was washed in PBS and sonicated to disperse cells. 10 µl of 5 x 10^6^ yeast cells/mL were loaded into 28 mm x 120 µm M04S microfluidic CellASIC cell culture plates (Millipore) in HMM (containing 110 mM glucose) or HMM/100 mM GlcNAc (containing 100 mM GlcNAc and 10 mM glucose) medium and transferred to RT. GFP fluorescence was measured with a 488 nm laser and DIC images were taken as described in the main text using confocal microscopy on live cells at each indicated timepoint.

**Morphology assessment of *C. albicans* Hc*NGT1* and Hc*NGT2* strains**

*H. capsulatum* *NGT1* (Hc*NGT1*), *H. capsulatum* *NGT2* (Hc*NGT2*), *C. albicans NGT1* (Ca*NGT1*), or empty vector (EV) were introduced into *C. albicans* *ngt1*Δ yeast cells (*ngt1*Δ) as described in the main text Materials and Methods. Yeast-form cells of each transformant were grown at 30°C overnight. The morphology of transformants was examined by light microscopy using DIC microscopy after 3 hours of growth at 37°C in minimal Yeast Nitrogen Base liquid medium containing 2.5 mM glucose or 2.5 mM GlcNAc and compared to the morphology of wild-type *C. albicans* (WT).

**Influence of chitin on *H. capsulatum* morphogenesis at RT**

Yeast form cultures of G217B were grown to early log phase at 37°C in HMM medium, washed once in PBS, and sonicated for 3 s to disperse clumps. 10 µl of G217B 5 x 10^6^ yeast cells/mL were loaded into 28 mm x 120 µm M04S CellASIC microfluidic cell culture plates (Millipore) in HMM (110 mM glucose), HMM supplemented with 500 ng/mL GlcNAc, and HMM supplemented with 500 ng/mL Chitin (Crab Shell Chitin, Sigma-Aldrich) media and transferred to RT. Chitin was prepared by resuspension in sterile water at approximately 1 mg/mL followed by sonication with a tip sonicator to form a cloudy suspension. At each indicated timepoint, cell morphology was examined using live-cell DIC confocal microscopy as described in the main text Materials and Methods.

**Supplementary References**

1. Nguyen VQ, Sil A (2008) Temperature-induced switch to the pathogenic yeast form o*f Histoplasma capsulat*um requires Ryp1, a conserved transcriptional regulator. Proc Natl Acad Sci U S A 105: 4880-4885.
